# Screening, identification and evaluation of an acidophilic strain of *Bacillus velezensis* B4-7 for the biocontrol of tobacco bacterial wilt

**DOI:** 10.3389/fpls.2024.1360173

**Published:** 2024-05-01

**Authors:** Xiang-jia Meng, Lan-qin Wang, Bai-ge Ma, Xi-hong Wei, Yi Zhou, Zheng-xiang Sun, Yan-yan Li

**Affiliations:** ^1^ College of Agriculture, Yangtze University, Jingzhou, Hubei, China; ^2^ Early Detection and Management of Agricultural and Forestry Pests, Jingzhou, Hubei, China; ^3^ Tobacco Research Institute of Hubei Province, Wuhan, Hubei, China

**Keywords:** tobacco bacterial wilt, microbiome, biocontrol, suppressive soil, *Bacillus velezensis*

## Abstract

Tobacco (*Nicotiana tabacum* L.) bacterial wilt, caused by *Ralstonia solanacearum*, is indeed a highly destructive plant disease, leading to substantial damage in tobacco production. While biological control is considered an effective measure for managing bacterial wilt, related research in this area has been relatively limited compared to other control methods. In order to discover new potential antagonistic bacteria with high biocontrol efficacy against tobacco bacterial wilt, we conducted an analysis of the microbial composition differences between disease-suppressive and disease-conducive soils using Illumina sequencing. As a result, we successfully isolated six strains from the disease-suppressive soil that exhibited antibacterial activity against *Ralstonia solanacearum*. Among these strains, B4-7 showed the strongest antibacterial activity, even at acidic conditions with a pH of 4.0. Based on genome analysis using Average Nucleotide Identity (ANI), B4-7 was identified as *Bacillus velezensis*. In greenhouse and field trials, strain B4-7 significantly reduced the disease index of tobacco bacterial wilt, with control efficiencies reaching 74.03% and 46.88% respectively. Additionally, B4-7 exhibited plant-promoting abilities that led to a 35.27% increase in tobacco production in field conditions. Quantitative real-time (qPCR) analysis demonstrated that strain B4-7 effectively reduced the abundance of *R. solanacearum* in the rhizosphere. Genome sequencing and Liquid Chromatography-Mass Spectrometry (LC-MS) analysis revealed that strain B4-7 potentially produces various lipopeptide metabolites, such as microlactin, bacillaene, difficidin, bacilysin, and surfactin. Furthermore, B4-7 influenced the structure of the rhizosphere soil microbial community, increasing bacterial abundance and fungal diversity, while also promoting the growth of different beneficial microorganisms. In addition, B4-7 enhanced tobacco’s resistance to *R. solanacearum* by increasing the activities of defense enzymes, including superoxide dismutase (SOD), phenylalanine ammonia-lyase (PAL), peroxidase (POD), catalase (CAT), and polyphenol oxidase (PPO). Collectively, these findings suggest that *B. velezensis* B4-7 holds significant biocontrol potential and can be considered a promising candidate strain for eco-friendly management of tobacco bacterial wilt.

## 1 Introduction

Tobacco bacterial wilt (TWB) is a highly destructive disease caused by *Ralstonia solanacearum*, resulting in significant economic losses in tobacco production annually (Jiang et al., 2017). Several factors influence the occurrence of TWB, with soil environmental conditions being of utmost importance. These conditions include soil type, microorganism composition, pH levels, and trace element content (Ahmed et al., 2022). In recent years, China has experienced severe soil acidification in tobacco-growing areas due to factors such as continuous cropping, environmental pollution, and the use of acidic fertilizers. Furthermore, *R. solanacearum* thrives in acidic environments, exacerbating the problem of TWB (Li et al., 2017a). As a consequence, there is a strong correlation between soil acidification and the incidence of bacterial wilt, with significantly lower soil pH levels observed in affected areas compared to unaffected regions (Seo et al., 2012). The severity of bacterial wilt increases in acidified tobacco soil, leading to reduced tobacco yield and quality. Therefore, addressing soil acidification is a crucial step in managing TWB and mitigating its detrimental effects on tobacco cultivation.

Current measurements to control wilt disease are mostly through the resistant cultivars, the chemical pesticides, and agricultural management. However, these management techniques are inefficient, do not provide durable resistance for the plants (Yang et al., 2018; Wang et al., 2020a). Moreover, the use of chemical fungicides poses significant risks to human health and the natural environment, while also leading to the development of drug resistance in pathogenic bacteria (Wu et al., 2014; Liu et al., 2016; Nithya et al., 2019). Additionally, in many crops, cultivars that are resistant to bacterial wilt often exhibit reduced quality, and resistant cultivars may not achieve desired yields (Nion and Toyota, 2015; Nelson et al., 2018). To address these challenges, the use of biological control agents (BCAs) has gained widespread attention as a safer, more effective, and sustainable approach to control bacterial wilt (Hu et al., 2021). Consequently, development of new biocontrol strategies is evidently needed.

Nowadays, the use of disease suppressive biocontrol agents, such as endophytes and rhizobacteria, has emerged as an alternative measure to reduce the incidence of bacterial wilt (Elsayed et al., 2019; Yan et al., 2022). These BCAs provide broad-spectrum benefits to host plants, including the production of antimicrobial compounds, promotion of plant growth, competition with pathogens for nutrients (Li et al., 2019), modulation of the rhizosphere soil bacterial community (Wu et al., 2016; Li et al., 2021), and induction of plant immune responses (Wang et al., 2020b; Wei et al., 2021). Notably, the biocontrol of bacterial wilt has been investigated with practical field applications using antagonistic species such as *Bacillus* (Wu et al., 2020; Tian et al., 2023), *Burkholderia* (Carrión et al., 2018), *Pseudomonas* (Ma et al., 2018), *Streptomycete* (Kaari et al., 2022), *Penicillium* (Dey et al., 2022), *Trichoderma* (Yuan et al., 2016), Bacteriophage (Elhalag et al., 2018) and Pathogen mutants (Li et al., 2022). Additionally, the combined use of multiple bacteriostatic strains has shown higher efficacy in biocontrol of bacterial wilt compared to single strains (Yu et al., 2022). However, the majority of microorganisms fail to establish a stable biocontrol effect in acidic soil environments (Rousk et al., 2010). Therefore, there is a need to identify biocontrol bacteria that can effectively survive and reproduce in acidic soils, ensuring their sustainable impact.

The plant rhizosphere community is particularly crucial for plant growth and health, playing a vital role in regulating soil fertility, nutrient cycling, and protecting plants against diseases (Wang et al., 2017). Soils that exhibit low disease incidence, even when susceptible plants and pathogens coexist, are referred to as disease-suppressive soils (SS), while the corresponding soils that are prone to disease are called disease-conducive soils (CS) (Weller et al., 2002; Qi et al., 2019; Zhang et al., 2020a). It has been observed that there are significant differences in soil composition between suppressive and conducive soils, despite similar geographical, climatic conditions, and agronomic management practices (Zheng et al., 2021). Studies have revealed that bacterial networks in suppressive soils are more complex and stable compared to conducive soils. In contrast to suppressive soils, conducive soils exhibit significantly reduced abundance and diversity of microorganisms (Wei et al., 2019; Lee et al., 2020; Zheng et al., 2021). Therefore, it is hypothesized that it may be easier to isolate strains with strong antibacterial effects against tobacco bacterial wilt in suppressive soils. For example, Zheng et al. (2021) isolated three *Pseudomonas* strains from suppressive soil, showing strong inhibitory effects on pathogenic bacteria under greenhouse conditions and reducing the incidence of bacterial wilt.

In this study, we conducted an investigation on two contrasting fields with regards to bacterial wilt disease. Despite being geographically close and managed similarly, the disease incidence was observed to be 0% in the suppressive soil while it was 100% in the conducive soil. We hypothesized that these differences were attributed to variations in bacterial community structure, as well as the presence of keystone taxa in the suppressive soil. Our aims were as follows: a) To examine the differences in bacterial community structure between the suppressive soil and the conducive soil; b) To identify the keystone taxa present in the suppressive soil; c) Isolation, screening, and identification of acidophilic bacteria with antagonistic properties against *R. solanacearum* in the suppressive soil; d) To evaluate the efficacy of the selected biocontrol agent (BCA) against TBW under greenhouse and field conditions; e) To assess the impact of the BCA on the tobacco rhizosphere microbiota through Illumina MiSeq sequencing of 16S rRNA gene amplicons; f) To analyze the potential of the selected BCAs to produce antibacterial metabolites by performing genomic sequencing and Liquid Chromatography-Mass Spectrometry (LC-MS); g) To determine the effects of the BCAs on the activity of tobacco defense-related enzymes.

## 2 Materials and methods

### 2.1 PCR amplification and high-throughput sequencing of soil bacteria

Soil samples were obtained from tobacco fields in Xuan’en County, Hubei Province (29.97°N, 109.38°E). These fields had a history of continuous cultivation of tobacco (Yunyan 87) for more than 15 years. Each area consisted of one block of suppressive soil (SS) and one block of conducive soil (CS). The rhizosphere soil of tobacco plants was collected using the five-point sampling method, 70 days after tobacco transplantation. A total of 30 soil samples were collected from the different areas. After collection, the samples were mixed thoroughly and transported back to the laboratory on dry ice to maintain their freshness. Subsequently, the samples were stored in a -80°C freezer for further experiments, including PCR amplification and high-throughput sequencing of soil bacteria. The rhizosphere soil was sent to Majorbio Gene Co., Ltd (Shanghai municipality, China) for sequencing analysis. Total DNA was extracted from the soil using the DNeasy PowerSoil Pro Kit, and this DNA was used as a template for further analysis (The raw data has been uploaded to NCBI, https://www.ncbi.nlm.nih.gov/, BioProject Number: PRJNA1089359). The specific sequencing methods and data processing are in the **Supplementary Materials and Methods S1**

### 2.2 Isolation of antagonistic strains

First, a 10g sample of field tobacco rhizosphere soil was weighed and placed in a triangular bottle containing 90 mL of sterile water. The mixture was then shaken at 28°C and 150 r/min for 20 minutes, resulting in the preparation of a soil suspension stock (Cui et al., 2020). This stock was further diluted to different concentrations (10^-2^, 10^-3^, 10^-4^, 10^-5^) using a 10-fold gradient. Next, 100 μL of the soil suspensions at different concentrations were absorbed and coated onto 2,3,5-triphenyl tetrazolium chloride (TTC) agar (Kelman, 1954), which had been pre-mixed with *R. solanacearum* (GenBank: OR711027) obtained from the Research Laboratory of Plant-Microbial Interaction in the College of Agriculture, Yangtze University, Jingzhou, China. Each concentration was treated three times to ensure accuracy and reliability. The coated plates were incubated in the dark at 28°C for 2 days. After incubation, the plates were examined for the presence of an inhibitory zone. BCAs that exhibited inhibitory activity, as indicated by the presence of an inhibitory zone, were selected as the test strains for further investigations.

### 2.3 Screening of antagonistic strain in eosinophilic rhizosphere

First, the pH of the nutrient broth (NB) was titrated to 4.0, 5.0, 6.0, and 7.0 using an acid-base buffer. The medium was then sterilized at 121°C for 20 minutes. Next, the antagonistic strains were inoculated into NB with different pH values, while *R. solanacearum* was inoculated into NB (pH 7.0). The cultures were incubated with shaking at 28°C and 150 r/min for 48 h. The concentration of BCAs and *R. solanacearum* fermentation broth was adjusted to 10^8^ CFU/mL. To assess the inhibitory effect of the fermentation broth on *R. solanacearum*, 200 µL of *R. solanacearum* fermentation broth was mixed with 100mL (50°C) of TTC medium using the agar diffusion method (Ma et al., 2018). The mixture was thoroughly mixed and poured into plates. Using a hole puncher, a 5 mm diameter hole was made in the center of each plate. Each hole was then filled with 200 µL of antagonistic bacterial fermentation broth. This process was repeated three times for each treatment, with an equal amount of NB used as a control. The plates were incubated at 28°C for 48 h, and the diameter of the inhibition zone was measured. The width of the inhibition zone determined the inhibitory effect of the antagonistic bacterial fermentation broth at different pH levels on *R. solanacearum*.

To further investigate the antibacterial activity of the antagonistic strains, the fermentation filtrate (FL) was tested for its inhibitory effect under acidic conditions. The fermentation broths of the antagonistic strains, prepared as described earlier with different pH values, were centrifuged at 8000 r/min for 5 minutes at 4°C. The resulting supernatant was then filtered through a microporous filter (0.22 mm) to remove any bacterial cells, obtaining a sterile filtrate. The inhibitory effect of the FL on *R. solanacearum* was determined by measuring the width of the inhibition zone, following the same procedure mentioned earlier for the fermentation broth bacteriostatic method.

### 2.4 Biocontrol activity of B4-7 against TBW in greenhouse pot

Tobacco seeds (Yunyan 87) were planted in plastic pots filled with tobacco field soil in Xuan’en County, Enshi City, Hubei Province. Once the tobacco seedlings reached the 3-leaf stage and showed consistent growth, they were transplanted into new plastic pots and continued to be cultivated until the 6-8 leaf stage for experimental treatment. The B4-7 fermentation broth was diluted with NB to concentrations of 1×10^8^ CFU/mL (P_3_), 1×10^7^ CFU/mL (P_2_), and 1×10^6^ CFU/mL (P_1_). Then, 20 mL of the B4-7 fermentation broth was inoculated into the rhizosphere of the seedlings using the root irrigation method. Each treatment consisted of 30 tobacco plants, and the experiment was repeated 3 times. As a positive control, 20 mL of Streptomycin (100 mg/L, Solarbio) was inoculated, while 20 mL of NB was used as a negative control. Two days after the application of the B4-7 fermentation broth, 20 mL of *R. solanacearum* fermentation broth (1×10^8^ CFU/mL) was irrigated in the rhizosphere of the tobacco seedlings. All the tobacco seedlings were then placed in a greenhouse with a relative humidity of 70-80% and a temperature of 30 ± 2°C for further cultivation. After 14 and 21 days of inoculation with *R. solanacearum*, the disease severity of the TBW was assessed based on the rating scale developed by Li et al. (2020). Disease index (DI) was calculated using the formula DI = Σ(r × n)/(N × 9)×100. Here, ‘r’ represents the rating scale of disease severity, ‘n’ represents the number of infected tobacco plants with a rating of ‘r’, and ‘N’ represents the total number of tobacco plants tested. To determine the biocontrol efficiency (BE) of the treatment, the formula [(Disease index of control - Disease index of treated group)/Disease index of control]×100% was used.

### 2.5 Growth promotion for tobacco of B4-7 in greenhouse

This study aimed to determine the growth promotion effect of antagonistic strains on tobacco under indoor pot conditions. Tobacco field soil was mixed with the fermentation broth B4-7 to achieve bacterial concentrations of 1.0×10^8^ CFU/g (Q_3_), 1.0×10^7^ CFU/g (Q_2_) and 1.0×10^6^ CFU/g (Q_1_). The tobacco seedlings were cultivated using the described method until they reached the three-leaf stage, and seedlings with the same size and growth were selected for transplantation. Each treatment was replicated three times, with each replicate consisting of 20 tobacco seedlings. Soil mixed with NB served as the control, while *Bacillus amyloliquefaciens* YZU-SG146 was used as a positive control (Liu et al., 2023). The tobacco seedlings were placed in a greenhouse with a relative humidity of 70~80% and a temperature of 30 ± 2°C for continuous cultivation, and their growth was monitored daily. Measurements of growth parameters, including plant height, root length, fresh weight, dry weight, stem diameter, and chlorophyll content (Chlorophyll analyzer TYS-3N; Beijing Zhongke Weihe Technology Development Co., LTD), were recorded after 10, 20, and 30 d.

### 2.6 Biocontrol activity of B4-7 against TBW in the field

The experiment was conducted in Xuan ‘en County, Enshi City, Hubei Province, at a tobacco field severely affected by bacterial wilt (29.97°N, 109.38°E). The experimental site consisted of three blocks, each covering an area of 260 m^2^. Within each block, there were two plots measuring 130 m^2^ each, following a completely randomized block design. One plot represented the B4-7 treatment, while the other served as the control. Tobacco plants were grown in a monocropping system, with a spacing of 0.55 m between plants within a row and 1.2 m between rows. Healthy tobacco seedlings were cultivated in a greenhouse until they reached the nine-leaf stage, after which they were transplanted into the field. The seedlings were inoculated with B4-7 fermentation broth, containing a concentration of 1×10^8^ CFU/mL, at 30 and 45 d after transplanting. Each plant was irrigated with 100 mL of the B4-7 fermentation broth, while the control group received NB as the irrigation solution. At 60 and 75 d after transplanting, the severity of TBW was assessed using a disease severity rating scale, and the disease index was calculated accordingly referred to Section 2.5. After the tobacco plants were harvested, yield data were collected for all plots. The actual yield of each plot was determined by weighing the dry tobacco leaves.

### 2.7 Genomic sequencing and functional annotation of strain B4-7

Strain B4-7 was activated on nutrient agar (NA) plate, inoculated into 50 mL NB, incubated at 28°C at 130rpm for 24h, and sent to Benagen corporation (Wuhan, Hubei, China) for whole genome sequencing (GenBank ID: CP080760). The experimental process was carried out according to the standard protocol provided by Oxford Nanopore Technologies (ONT), including sample quality inspection, library construction, library quality inspection and library sequencing. Sequencing was performed using Next-Generation sequencing (NGS) from the Illumina MiSeq sequencing platform and real-time electrical signal sequencing from Nanopore sequencing technology. After filtering the raw data, short fragments and low quality data, the filtered reads were assembled using unicycler(0.4.8) software. The prediction of coding genes was carried out by prokka software, which used prodigal to predict coding genes, including tRNA, rRNA, miscRNA, and the preliminary annotation was completed after the summary of various predicted genetic elements. The Genome BLAST Distance Phylogeny approach (GBDP) between B4-7 and homologous strains was calculated based on the B4-7 16S rDNA gene (GenBank ID: OM370806), and a phylogenetic tree was constructed using FastME 2.1.6.1 software. digital DNA-DNA hybridization (dDDH) values of B4-7 and other homologous strains were calculated using GGDC3.0 software. Through online services JSpeciesWS platform (https://jspecies.ribohost.com/jspeciesws/#home) B4-7 compare with other genome Average Nucleotide Identity (ANI) (Chun et al., 2018). The predicted gene sequences were BLAST compared with the functional databases of Gene Ontology (GO), Kyoto Encyclopedia of Genes and Genomes (KEGG) and Clusters of Orthologous Group (COG) to obtain the gene function annotation results (Ogata et al., 1999; Tatusov et al., 2000). Using circlize in the R package, the predicted genomic information, such as sequencing depth, GC distribution and genome structure annotation were mapped into the genome circle. Biosynthetic gene clusters (BGCs) were identified by the online antiSMASH software (Weber et al., 2015).

### 2.8 Effects of B4-7 on rhizosphere microorganisms of tobacco

At 60 and 75 days after transplanting, rhizosphere soil samples were collected from five separate sites within each plot to evaluate disease severity. A total of six soil samples, each weighing 150~200 g, were collected by mixing the samples from the five sites within each plot. Each set of soil samples was then divided into two sub-samples for further analysis. One sub-sample (only the soil samples from 75 days) was sent to Majorbio Gene Co., Ltd for sequencing analysis of fungi and bacteria (BioProject Number: PRJNA1093021). The specific sequencing methods and data processing are in the **Supplementary Materials and Methods S1**. The other sub-sample was used for quantitative real-time (qPCR) to quantify the presence of *R. solanacearum* by specific primers specific primer combination of 199F (5’-AGTAACTCGGCTGTTTTTTT-3’) and 199R (5’-TATTGCTTGACCTATAA-3’). The specific methods is in the **Supplementary Materials and Methods S2**.

### 2.9 Detection of defense-related enzyme activities in plants

After inoculating the tobacco plants with the *R. solanacearum* in a greenhouse, the third to fifth unfolded leaves of each plant were collected from top to bottom every 48 hours, for a total of three collections. The collected leaves were immediately stored at -80°C for further analysis. The activities of several defense enzymes, including phenylalanine ammonia-lyase (PAL), peroxidase (POD), catalase (CAT), polyphenol oxidase (PPO), and superoxide dismutase (SOD), in the leaves under each treatment were determined. For comparison, the treatments included inoculation with the pathogenic bacteria as a disease control group, inoculation with NB as a healthy control group. The extraction of the defense enzyme solution and the determination of its activity were conducted using a kit from Solarbio, following the instructions provided (Zhao et al., 2020).

### 2.10 Antibacterial activity, thermal stability and composition analysis of crude extract

1L FL of B4-7 was prepared referred to Section 2.3. After the supernatant of B4-7 was freeze-dried, resulting in a flocculent solid, the solid was dissolved by adding 100 mL of sterile distilled water to the mixture. The mixture was then transferred to a liquid separation funnel, and an equal amount of ethyl acetate was added. The mixture was shaken and mixed thoroughly. The mixture was left undisturbed to allow complete stratification. During this process, a clear separation occurred between the upper organic phase and the lower aqueous phase. The upper organic phase was carefully collected, while the lower aqueous phase was retained. The lower aqueous phase was subjected to further extraction by adding ethyl acetate. This extraction process was repeated three times, with each extraction carried out for approximately 12 hours. The organic phases obtained from the three extractions were combined, resulting in a merged organic phase. The merged organic phase was then concentrated using a rotary evaporator set at 50°C. The rotary evaporation process removed the solvent, leaving behind a dried crude extract. After the solvent has been completely removed, the dried crude extract was dissolved in a small amount of chromatograph-grade methanol. Finally, the dissolved crude extract was stored in a refrigerator at 4°C for future use.

After the FL of B4-7 was processed and the crude extract was obtained, the extract was further diluted with methanol to several concentrations: 50μg/mL, 100μg/mL, 200μg/mL, and 500μg/mL. To evaluate the antimicrobial activity of the crude extract, a TTC agar was prepared by adding 200µL of *R. solanacearum* fermentation broth into 100mL of TTC agar at 50°C. The medium was then cooled. Symmetrical holes with a diameter of 5 mm were created at a distance of 25mm from the center of the TTC medium plate. Each hole was injected with 150 µL of the different concentrations of the crude extract solutions. This procedure was repeated three times for each treatment. Methanol was used as a control. All the TTC plates, including the control, were transferred into a 28°C incubator. After two days of incubation, the diameter of the inhibition zones, which indicates the growth inhibition of pathogens, was measured using a vernier caliper.

In addition to evaluating the antimicrobial activity of the crude extract at different concentrations, further experiments were conducted to investigate the effect of different temperature treatments on the extract’s potency. The crude extract, prepared at a concentration of 500 μg/mL, was divided into several equal parts and subjected to various temperature treatments: (a) Water bath at 25°C for 30 min; (b) Water bath at 40°C for 30 min; (c) Water bath at 60°C for 30 min; (d) Water bath at 80°C for 30 min; (e) Water bath at 100°C for 30 min; (f) Treatment with a sterilized pot at 121°C for 30 min. Perforated plates were prepared using the same method as before, and each hole was injected with 150 μL of the crude extract solution that underwent a different temperature treatment. This process was repeated three times for each treatment. All the plates, including the control, were then incubated at 28°C. After two days of incubation, the diameter of the inhibition zones was measured using a vernier caliper.

The composition of lipopeptide-producing active substances of strain B4-7 was analyzed using high-resolution liquid chromatography-mass spectrometry (LC-MS). The specific methods is in the **Supplementary Materials and Methods S3**.

## 3 Results

### 3.1 Rhizosphere bacterial community of suppressive soil and conducive soil

**Figure 1:**
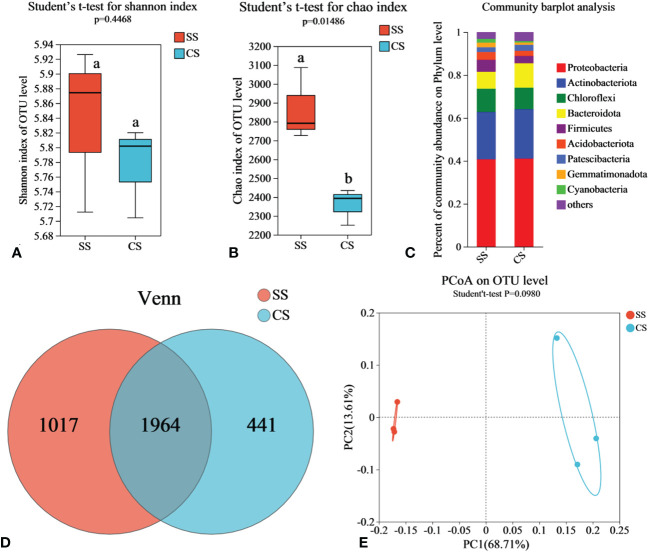
Soil bacterial community in disease-suppressive soil (SS) and disease-conducive soil (CS). Shannon diversity **(A)** and Chao richness **(B)** indices of bacteria community in rhizosphere soils are presented as the mean. Different lowercase letters (Student’ s t-test, p < 0.05) indicate statistically significant differences between two groups. **(C)** The relative abundance of bacterial phylum level in soil samples. **(D)** Venn diagrams based on total taxa at the bacterial OTUs level. **(E)** The principal co-ordinates analysis (PCoA) and Beta diversity difference analysis. (Student’ s t-test, p < 0.05) indicates significant diversity between groups.

To compare the bacterial communities between suppressive soil (SS) and conducive soil (CS), rhizosphere soil samples were collected from fields in Enshi county. Illumina MiSeq sequencing was conducted to analyze the soil microbiome. Rarefaction curve analysis was performed to evaluate the reasonableness of the sequencing depth. As shown in **Supplementary Figure S1A**, although the curve did not reach a final plateau, it flattened out when the sequencing depth reached 40,000, indicating that increasing the sequence number would not result in the emergence of new Operational Taxonomic Units (OTUs). The sequencing depth was sufficient to represent the microbial community in these environments. According to Venn analysis, there were 1,964 bacterial OTUs shared by both SS and CS, with 1,017 endemic OTUs found in SS and 441 endemic OTUs in CS. The number of endemic OTUs in SS was 2.31 times higher than that in CS (**Figure 1D**). According to Shannon and Chao indices, the richness of bacteria in SS increased significantly compared with CS, but there was no significant difference in diversity (**Figures 1A, B**). Based on the results of the PCoA with the Bray-Curtis measures, SS and CS explained 82.32% of the total bacterial community variation in PC1 and PC2. Although there was significant separation on PC1 axis, combined with Beta diversity difference analysis. (Student′s t-test, p < 0.05), there was no significant difference in species diversity between SS and CS (**Figure 1E**).

The relative species abundance at the taxonomic level of SS and CS phylum is shown in **Figure 1C**, the dominant microflora at the phylum level in SS includes Proteobacteria (40.59%), Actinobacteriota (22.11%), Chloroflexi (10.92%), Bacteroidota (7.82%), Firmicutes (5.59%), Acidobacteriota (3.63%), Patescibacteria (2.23%), Gemmatimonadota (2.17%) and Cyanobacteria (1.79%). In CS, the dominant microflora at the phylum level consists of Proteobacteria (41.1%), Actinobacteriota (22.77%), Chloroflexi (9.97%), Bacteroidota (11.56%), Firmicutes (3.34%), Acidobacteriota (2.47%), Patescibacteria (2.74%) and Gemmatimonadota (1.07%). These results indicate clear differences in the abundance of Bacteroidota, Acidobacteriota, Firmicutes, and Gemmatimonadota between SS and CS. Compared to CS, the relative abundance of Firmicutes, Acidobacteriota, and Gemmatimonadota in SS increased by 67.19%, 46.52%, and 108.16%, respectively. The relative abundance of Bacteroidota in SS decreased by 32.38%. In the Heatmap (**Supplementary Figure S2**), the relative abundance of soil bacterial communities is depicted using a color gradient from red to white to blue, indicating high to low abundance, respectively. Compared with CS, the abundance of *Sphingobacterium* (p < 0.05), *Bacillus* (p < 0.001), *Streptomyces* (p < 0.05), *Chujaibacter* (p < 0.01), and *Sporosarcina* (p < 0.001) increased significantly, while the abundance of *Ralstonia* (p < 0.05), *Chryseobacterium* (p < 0.01), *Achromobacter* (p < 0.05), *Pseudomonas* (p < 0.001), and *Paenibacillus* decreased significantly.

### 3.2 Isolation, screening of acidophilic antagonistic strain against *R. solanacearum*

**Figure 2:**
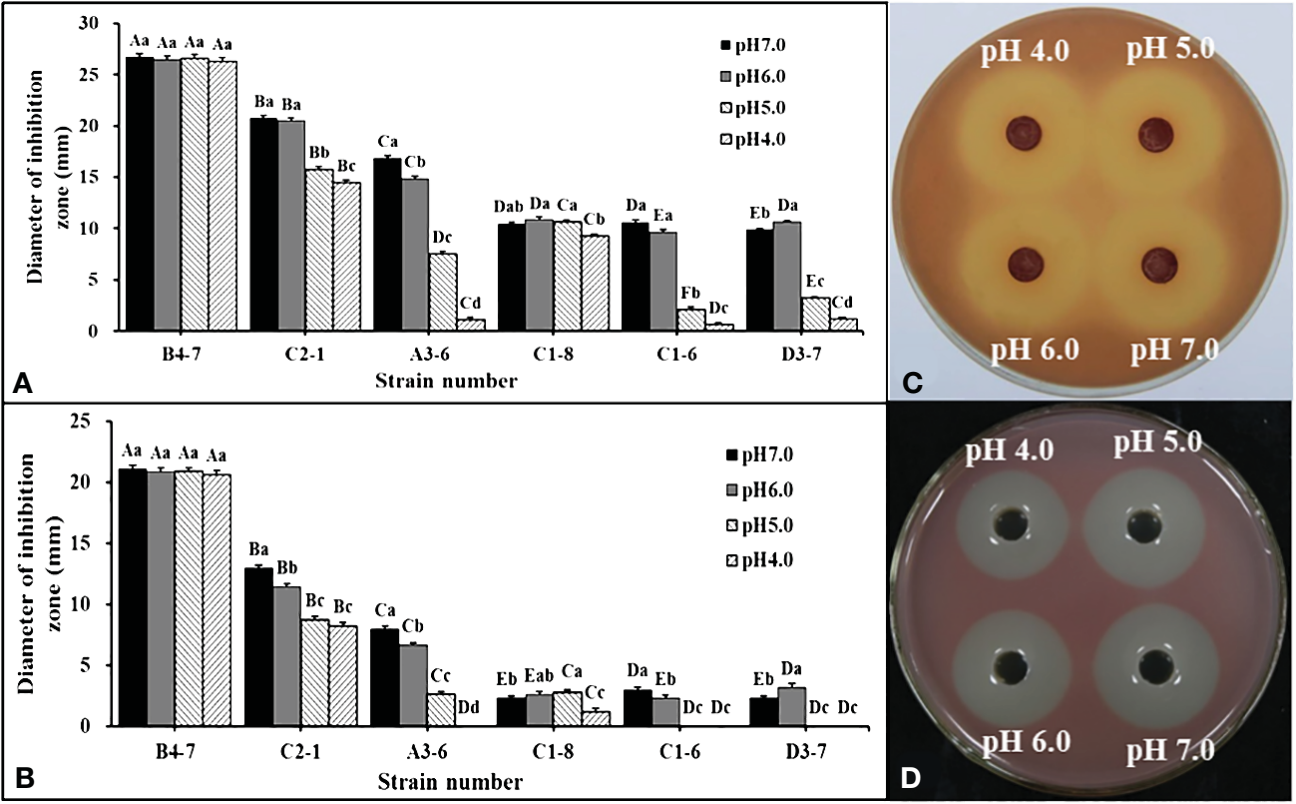
Inhibition effects of antagonistic bacteria against Ralstonia solanacearum at different pH. Inhibitory activity of bacterial fermentation broth **(A)** and fermentation filtrate (FL) **(B)** of six strains against R. solanacearum at different pH. Inhibitory activity of B4-7 fermentation broth **(C)** and FL **(D)** against R. solanacearum at different pH. The capital letters in **(A, B)** indicate the significant difference in antibacterial activity of different strains at the same pH, same letter indicate significant differences in antibacterial activity of the same strain at different pH. Bars indicate the standard error of the mean. Columns marked with the same letter are not significantly different according to Duncan’s Multiple Range Test at P < 0.05.

Forty-five isolates were obtained from disease-suppressive soil. Among these isolates, only six of them showed inhibitory effects on *R. solanacearum*. These six isolates were then inoculated into NB with different pH values to examine the effects of bacterial fermentation broth and fermentation filtrate (FL) on *R. solanacearum* in a dual-culture experiment. It was observed that compared to the other five strains, the isolate B4-7 fermentation broth exhibited the strongest antibacterial ability under different pH conditions (**Figure 2A, B**). Further analysis of the data revealed that under the conditions of pH 4.0, pH 5.0, pH 6.0, and pH 7.0, the inhibitory band widths of the fermentation broth of B4-7 against *R. solanacearum* were measured to be 26.36 mm, 26.61 mm, 26.48 mm, and 26.70 mm, respectively (**Figure 2C**). Similarly, the inhibitory band widths of the FL of B4-7 against *R. solanacearum* under these pH conditions were recorded as 20.61 mm, 20.84 mm, 20.87 mm, and 21.08 mm, respectively (**Figure 2D**). Furthermore, it was observed that under different pH conditions, the antibacterial effects of B4-7 fermentation broth and FL on *R. solanacearum* were not significantly different.

### 3.3 Biocontrol effects of *B. velezensis* B4-7 on TBW under pot

In the greenhouse experiment, tobacco plants were inoculated with the *R. solanacearum* and treated with different treatments including B4-7 fermentation broth and streptomycin. After 14 d of inoculation with *R. solanacearum*, the disease index of tobacco plants treated with B4-7 fermentat broth (at concentrations P_1_, P_2_, and P_3_) ranged from 13.32 to 20.88. On the other hand, the disease index of tobacco plants treated with streptomycin was 10.08. The control effects of the B4-7 fermentation broth ranged from 43.69% to 72.82%, indicating a reduction in disease severity compared to the control group. After 21 days of inoculation, the disease index of tobacco plants treated with B4-7 fermentation broth ranged from 18.36 to 35.34. The disease index of tobacco plants treated with streptomycin was 16.92. The control effects of the B4-7 fermentation broth ranged from 45.30% to 74.03%, again showing a reduction in disease severity compared to the control group. Specifically, when the concentration of B4-7 fermentation broth was 1×10^8^ CFU/mL, there was no significant difference in its control effect compared to streptomycin (**Table 1**; **Supplementary Figure S3**).

**Table 1:** Pot efficacy of strain B4-7 against tobacco bacterial wilt.

Treatment	14days		21days	
	Disease index^1,2^	Biocontrol efficacy(%)	Disease index^1,2^	Biocontrol efficacy(%)
B4-7(P_1_)	20.88±2.48b	43.69%	35.64±3.42b	45.30%
B4-7(P_2_)	15.84±1.93c	57.28%	23.04±2.22c	64.64%
B4-7(P_3_)	13.32±1.43d	64.08%	18.36±1.93d	71.82%
Streptomycin	10.08±1.24e	72.82%	16.92±1.26d	74.03%
CK	37.08±3.60a	–	65.16±5.37a	–

P_1_:10^6^CFU/mL. P_2_:10^7^CFU/mL. P_3_:10^8^CFU/mL. 1: Numerical values were mean ± SD of triplicates. 2: Means were tested using Duncan’s Multiple Range Test of SPSS 17.0 software. Means followed by the same letter are not significantly different (P < 0.05) within the same column.

### 3.4 Growth-promoting effects of *B. velezensis* B4-7 on tobacco seedlings

B4-7 can substantially promote the growth of tobacco seedlings (**Supplementary Figure S4**). When different concentrations of B4-7 fermentation broth (Q_3_, Q_2_, and Q_1_) were applied, the seedlings showed remarkable growth compared to the control. Specifically, the increases were as follows: plant height by 58.05%, 49.73%, and 8.76%; root length by 28.37%, 22.21%, and 2.17%; fresh weight by 36.93%, 23.48%, and 5.87%; dry weight by 86.36%, 59.09%, and 9.09%; stem diameter by 29.63%, 17.99%, and 2.65%; and chlorophyll content by 48.06%, 40.19%, and 14.12%, for Q_3_, Q_2_, and Q_1_ respectively. The growth indices of tobacco seedlings treated with B4-7 were found to be similar to those treated with YZU-G146, showing no significant difference (**Table 2**).

**Table 2:** Parameter of B4-7 promoting tobacco growth in greenhouse.

Growth parameter	B4-7 (Q_3_)	B4-7 (Q_2_)	B4-7 (Q_1_)	YZU-SG146	Control (NB)
Plant height/cm	14.43±1.08a	13.67±0.78a	9.93±0.32b	13.83±0.65a	9.13±0.65b
Root length/cm	18.96±2.42a	18.05±0.71ab	15.09±1.60b	17.97±1.47ab	14.77±1.55b
Fresh weight/g	7.23±0.27a	6.52±0.47b	5.59±0.23c	6.77±0.11ab	5.28±0.24c
Dry weight/g	0.41±0.04a	0.35±0.03b	0.24±0.02c	0.36±0.04ab	0.22±0.03c
Stem Diameter/mm	4.90±0.50a	4.46±0.42	3.88±0.16b	5.09±0.16a	3.78±0.47b
Chlorophyll content/SPAD	30.10±2.30a	28.50±2.01a	23.20±1.41b	28.40±1.57a	20.33±2.89b

Q_1_:10^6^CFU/g. Q_2_:10^7^CFU/g. Q_3_:10^8^CFU/g. 1: Numerical values were mean ± SD of triplicates. 2: Means were tested using Duncan’s Multiple Range Test of SPSS 17.0 software. Means followed by the same letter are not significantly different (P < 0.05) within the same line.

### 3.5 Biocontrol effects of *B. velezensis* B4-7 on TBW under field

**Figure 3:**
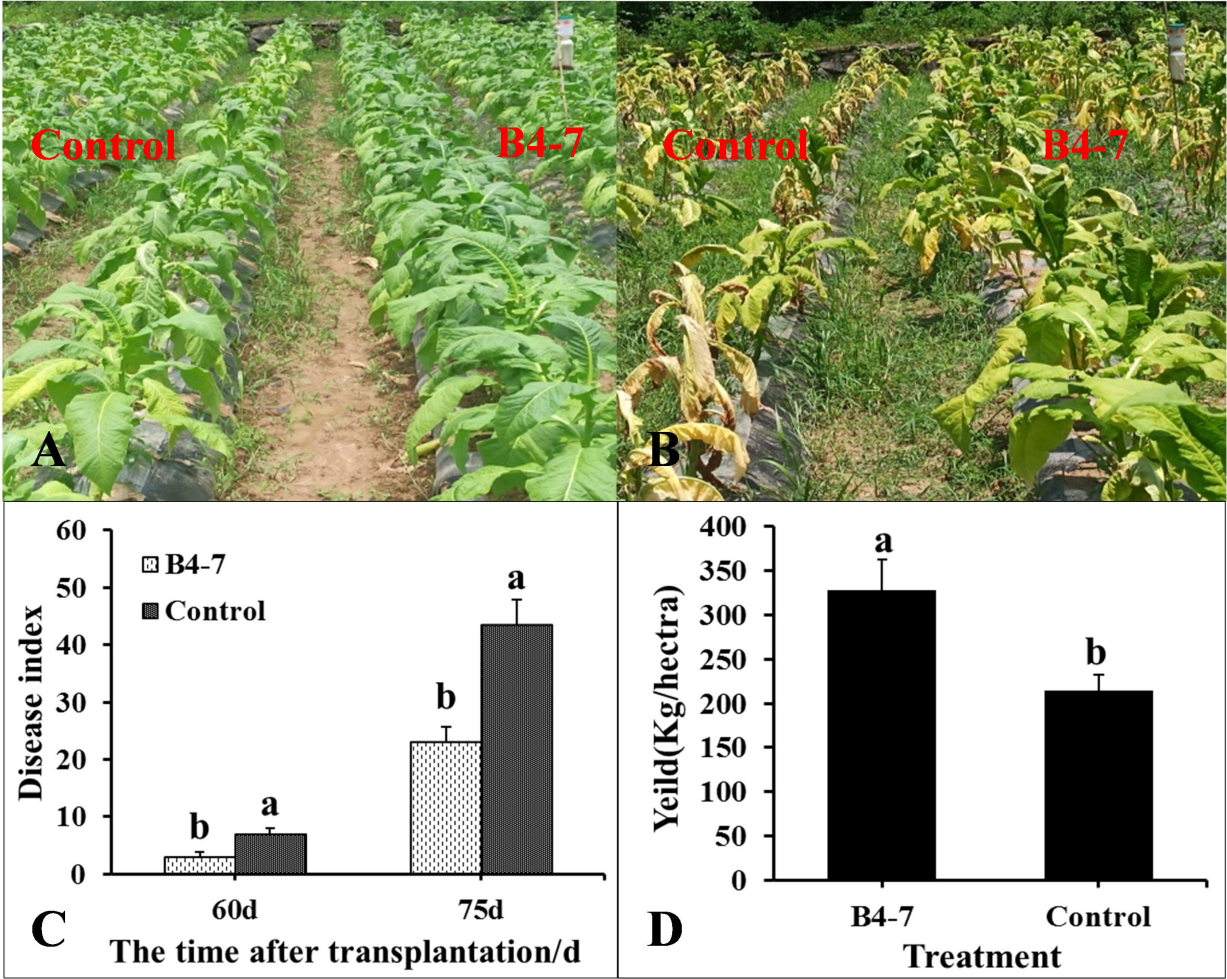
Control effect of B4-7 against TBW in Field. Phenotype of tobacco treated with B4-7 in fields at 60 d **(A)** and 75 d **(B)** after transplanting. **(C)** The disease index of TBW at different time of tobacco with different treatments. **(D)** Yield of tobacco under different treatments during the tobacco harvest. Bars indicate the standard error of the mean. Columns marked with the same letter are not significantly different according to Duncan’s Multiple Range Test at P < 0.05. Phenotype of tobacco treated with B4-7 in fields at 60 d **(A)** and 75 d **(A)** after transplanting. **(C)** The disease index of TBW at different time of tobacco with different treatments. **(D)** Yield of tobacco under different treatments during the tobacco harvest. Bars indicate the standard error of the mean. Columns marked with the same letter are not significantly different according to Duncan’s Multiple Range Test at P < 0.05.

**Figure 4:**
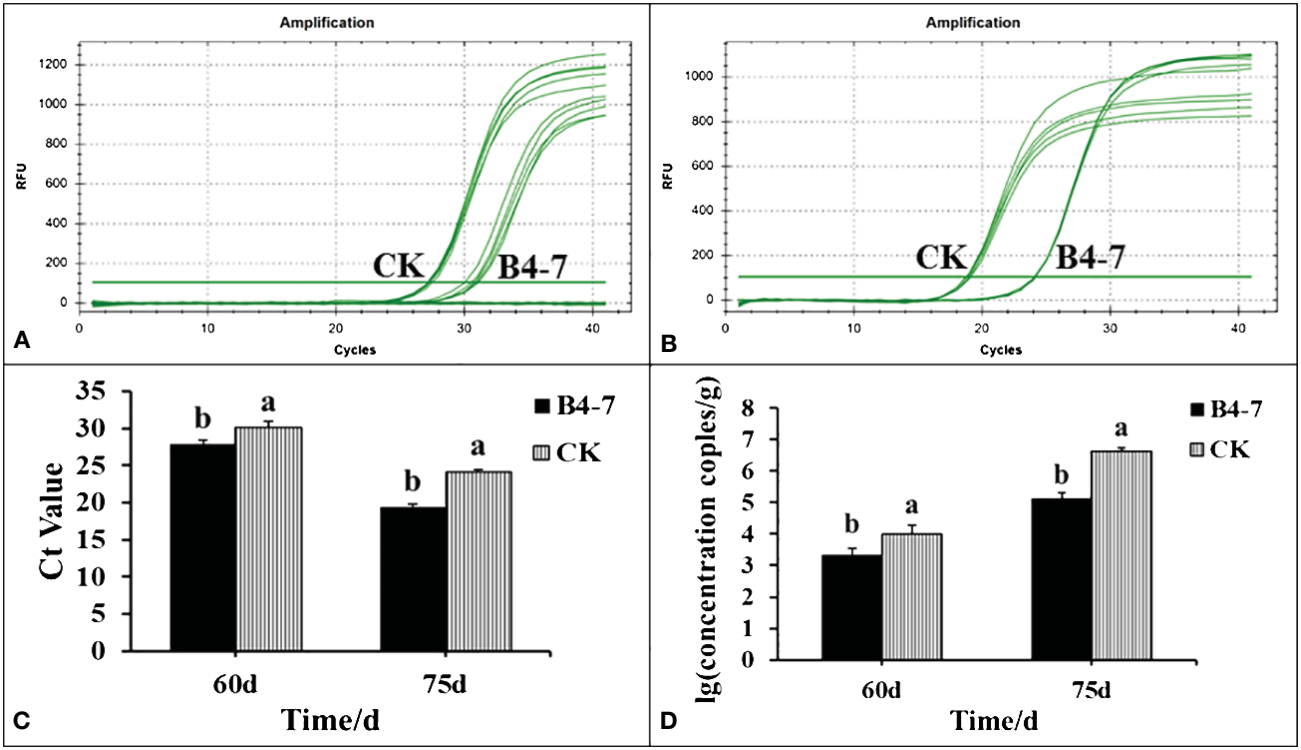
Amount of R. solanacearum in soil samples from B4-7 treatments. The rhizosphere soil of the two treatments was collected at 60 and 75 days after tobacco transplanting. Soil DNA was extracted and the number of pathogens in the rhizosphere was detected by RT-qPCR. RT-qPCR amplification curves of R. solanacearum DNA in rhizosphere 60 d **(A)** and 75 d **(B)** after transplanting. **(C)** RT-qPCR of R. solanacearum DNA Ct value in rhizosphere. **(D)** Number of R. solanacearum in tobacco rhizosphere.

The disease index and the quantity of rhizosphere *R. solanacearum* were evaluated after 60 and 75 days of tobacco transplantation. The *R*. *solanacearum* DNA amplification of tobacco rhizosphere was detected using qPCR, which has a sensitivity 1000 times greater than conventional PCR (**Supplementary Figures S5A** and **S5C**). The dissolution curves were unimodal (**Supplementary Figure S5B**), indicating no specific amplification at each concentration. The standard curve relating the concentration of *R. solanacearum* and the Ct value was established based on the amplification curve and used to calculate the concentration of *R. solanacearum* in the soil (**Supplementary Figure S5D**). After 60 days post transplantation, the disease index of TBW following B4-7 treatment was 3.14, biocontrol efficiency (BE) of 56.27% (**Figure 3**). Furthermore, the concentration of rhizosphere *R*. *solanacearum* was 2.07×10^3^ CFU/g, a drop of 78.81% in comparison to the control (**Figure 4**). After 75 days post transplantation, the disease index of TBW following B4-7 treatment was 23.18, with a control effect of 46.88% (**Figure 3**). The concentration of rhizosphere *R*. *solanacearum* stood at 1.32×10^5^ CFU/g, representing a reduction of 96.91% relative to the control (**Figure 4**). At the time of tobacco harvest, the yield of tobacco treated with B4-7 root irrigation was 326 kg/ha, representing an enhancement in yield by 35.27% compared to the control (**Figure 3**).

### 3.6 Genome sequencing and annotation of *B. velezensis* B4-7

After sequencing and assembly, the genome of *B. velezensis* B4-7 consisted of 3,926,832 bp and had 46.5% of GC content. The genome contained 27 rRNA genes, 86 tRNA genes, and 3743 coding sequences (**Supplementary Figure S6**). By using the Type Strain Genome Database (https://gctype.wdcm.org/), 13 strains homologous to B4-7 were obtained. The ANI value of B4-7 only exceeds 95% with that of *B.velezensis* FZB42 and *B. velezensis* NJN-6, indicating that B4-7 belongs to *B. velezensis* (**Supplementary Figure 7**). By annotation, 55.29%, 74.87% and 63.73% of genes were assigned to three categories of KEGG, GO, and COG, respectively. KEGG Pathway is the most commonly used KEGG database, and the number of genes matched by B4-7 in the KEGG pathway is 1177, accounting for 29.81%. The collection of metabolic pathways of B4-7 mainly includes five categories: cellular processes, environmental information processing, genetic information processing, melabalism and organismal systems (**Supplementary Figure S8**). Among them, 387 and 283 genes were involved in carbohydrate metabolism and amino acid metabolism, and 189 genes were involved in the metabolism of lipid, terpenoids and polyketides, as well as the biosynthesis of other secondary metabolites, including genes related to the biosynthesis of antibacterial active substances secreted by this strain. For COG annotation, the five groups with the largest proportion of genes are 263 genes involved in amino acid transport and metabolism [E], 202 genes involved in translation (ribosomal structure and biogenesis) [J], 199 genes involved in carbohydrate transport and metabolism [G], 183 genes involved in transcription [K], and 219 genes involved in general function prediction only [R]. Notably, 184 of genes were clustered into unknown function category [S] (**Supplementary Figure S9**). A total of 2956 genes were annotated into biological process (1,098), cellular component (2,168), and molecular function (1,989) using the GO database (**Supplementary Figure S10**).

**Figure 5:**
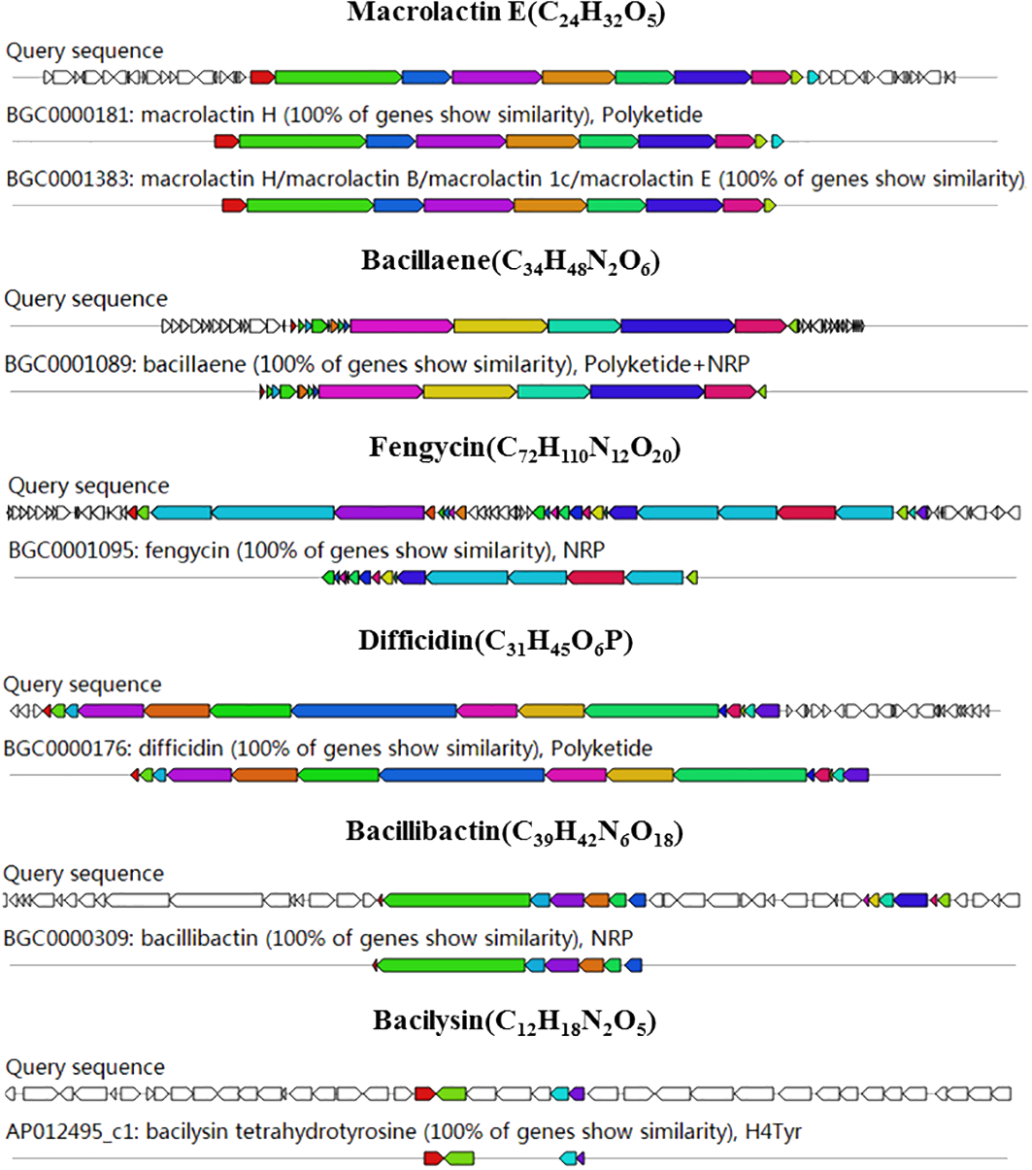
Genome annotation *B*. *velezensis* B4-7 and BGC prediction. Genomic information and chemical structure of BGCs with 100% similarity with the known BGCs.

The antiSMASH software was used to predict the gene clusters encoding secondary metabolites in the genome of strain B4-7. The predicted 12 BGCs included NRPS (non-ribosomal peptide synthetase), PKS (polyketide synthase), terpene, lanthipeptide-class-ii (**Supplementary Table S1**). Six BGCs showed more than 100% of similarity with macrolactin H, bacillaene, fengycin, difficidin, bacillibactin and bacilysin. One BGC showed more than 80% of similarity with surfactin (**Figure 5**). In addition, four unknown BGCs suggests that several novel secondary metabolites could be produced by strain B4-7.

### 3.7 Detection of antibacterial activity and composition of crude extract

**Figure 6:**
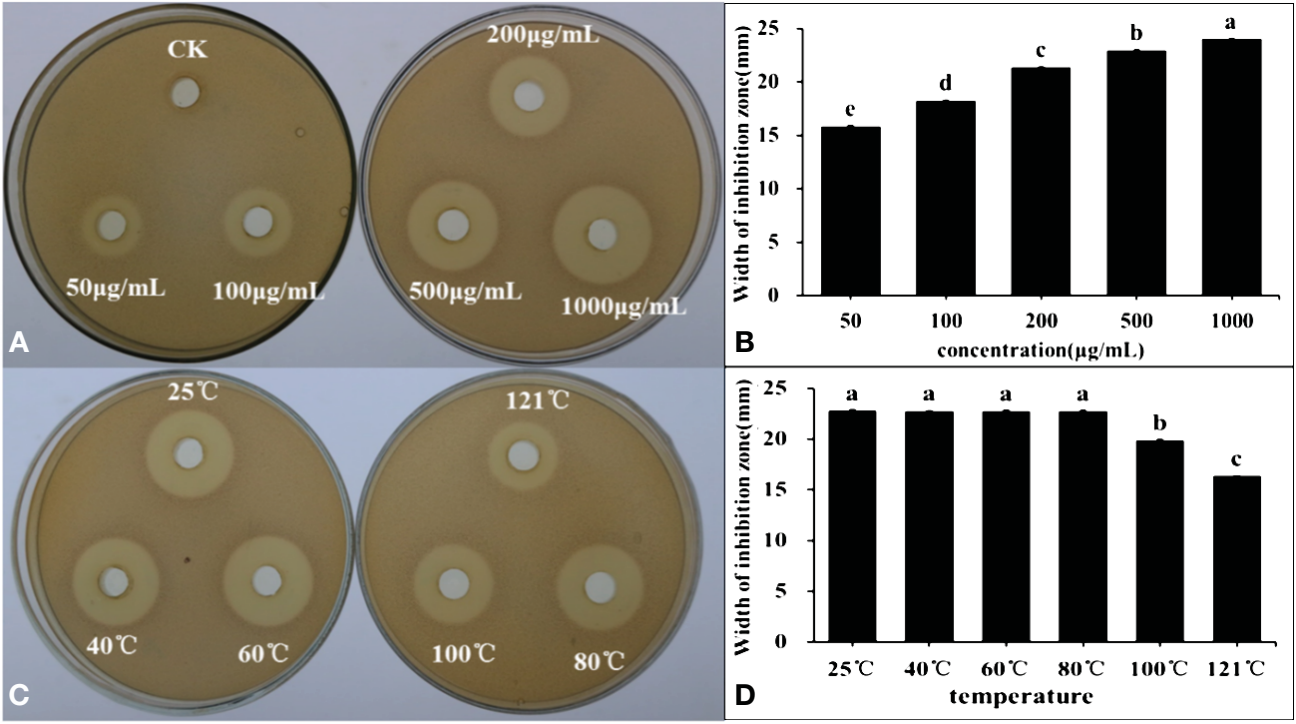
The inhibitory effect of crude lipopeptide against *R. solanacearum* extracted from FL of B4-7 **(A, B)**. The crude lipopeptides were regulated with methanol to 0(CK), 50, 100, 200, 500, and 1000 μg/mL, respectively, each at a concentration of 200 µL. The inhibitory effect of different temperature of B4-7 crude lipopeptide against R. solanacearum **(C, D)**. The crude lipopeptide were treated at 25°C, 40°C, 60°C, 80°C, 100°C and 121°C conditions for 30 minutes. The plates were incubated at 28°C for 2 days. Bars indicate the standard error of the mean. Columns marked with the same letter are not significantly different according to Duncan’s Multiple Range Test at P < 0.05.

Post freeze-drying, organic solvent extraction, and rotary evaporation, the lipopeptide active substances produced by the B4-7 strain were acquired. The antibacterial activity of these active substances was tested after they were dissolved in methanol. The findings revealed that the B4-7 strain’s lipopeptide active substances had robust antibacterial activity. The antibacterial bandwidths at concentrations of 50 μg/mL, 100 μg/mL, 200 μg/mL, 500 μg/mL, and 1000 μg/mL were 15.81 mm, 18.22 mm, 21.32 mm, 22.91 mm, and 24.02 mm, respectively (**Figure 6**). The crude extract’s concentration was adjusted to 500 µg/mL; it underwent temperature treatment by water bath heating and sterilization at 121°C. The crude extract’s antibacterial effects at various temperatures post treatment with the B4-7 active substance were tested using a plate drilling experiment. The results suggested that the antibacterial zone diameter of the crude extract did not markedly change when the temperature was set between 25°C and 80°C, averaging at 22.75 mm. However, when the temperatures were raised to 100°C and 121°C, the inhibition zone diameters were 19.87mm and 16.35mm, respectively (**Figure 6**).

The findings of the LC-MS analysis revealed that the crude extract of the B4-7 strain consisted of various organic compounds. Among these, the compounds with a high match degree were found to include microlactin (microlactin A, microlactin E), difficidin, bacilysin, surfactin (surfactin A, surfactin B, and surfactin C), bacillaene, Pachymic acid, Benzoylmesaconine, Troxerutin, and 3,4’,5-Trimethoxy-trans-stilbene, among others (**Table 3**; **Supplementary Figure S9**).

**Table 3:** Identification of compound components in B4-7 crude extract by LC-MS.

Peak time/min	molecular formula	Mass/ Charge	Ion binding form	Material type
5.931	C_31_H_43_NO_10_	590.3138	[M+H]^+^	Benzoylmesaconine
6.536~6.569	C_34_H_48_N_2_O_6_	581.3527	[M+H]^+^	bacillaene
7.527~7.561	C_24_H_32_O_5_	401.2324	[M+H]^-^	macrolactin E
7.817~7.850	C_24_H_34_O_5_	403.2485	[M+H]^+^	macrolactin A
8.276	C_33_H_42_O_19_	765.3799	[M+H]^+^	Troxerutin
5.524~5.557	C_31_H_45_O_6_P	545.2980	[M+H]^+^	difficidin
9.291~9.324	C_12_H_18_N_2_O_5_	271.1302	[M+H]^+^	bacilysin
10.412~10.445	C_51_H_89_N_7_O_13_	1008.6517	[M+H]^+^	surfactin A
10.913~10.946	C_52_H_91_N_7_O_13_	1022.6665	[M+H]^+^	surfactin B
11.193~11.226	C_53_H_93_N_7_O_13_	1036.6814	[M+H]^+^	surfactin C
12.118	C_17_H_18_O_3_	269.2698	[M-H]^-^	3,4',5-Trimethoxy-trans-stilbene
19.645	C_33_H_52_O_5_	527.4465	[M-H]^-^	Pachymic acid

### 3.8 Effects of *B. velezensis* B4-7 on the activity of tobacco defense-related enzymes

**Figure 7:**
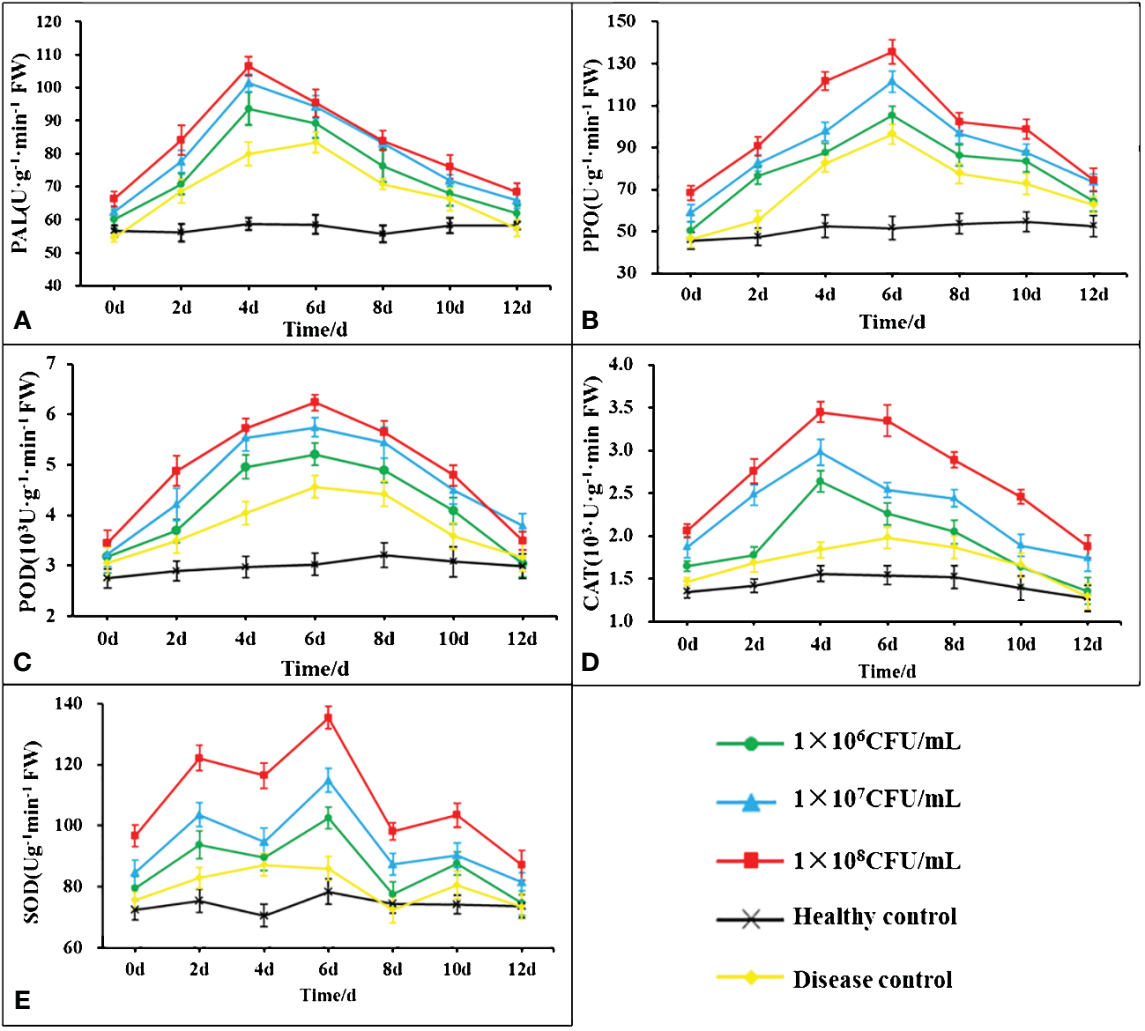
Effects of different concentrations of B4-7 bacterial suspension on defensive enzyme activities in tobacco at 0, 2, 4, 6, 8, 10, 12d after treatment. **(A)** Effects of different treatments on the activity of phenylalanine ammonia lyase (PAL); **(B)** Effects of different treatments on the activity of polyphenol oxidase (PPO); **(C)** Effects of different treatments on the activity of peroxidase (POD); **(D)** Effects of different treatments on the activity of catalase (CAT); **(E)** Effects of different treatments on the activity of superoxide dismutase (SOD). 1×106 CFU/mL: 1×106 CFU/mL B4-7 fermentation broth treatment; 1×107 CFU/mL: 1×107 CFU/mL B4-7 fermentation broth treatment; 1×108 CFU/mL: 1×108 CFU/mL B4-7 fermentation broth treatment; Healthy Control: NB treatment; Disease Control: pathogenic bacteria treatment.

The changes in the activities of POD, PPO, PAL, CAT and SOD content in the leaves of tobacco post *B. velezensis* B4-7 fermentation broth root treatment was showed in **Figure 7**. The POD, PPO, PAL and CAT showed a pattern initially increasing and then declining. The defense enzymes of different concentrations of B4-7 fermentation broth were significantly more substantial than those in the healthy and diseased controls. PAL and CAT activities peaked on the fourth day post-root irrigation, while POD, PPO and SOD activities peaked on the sixth day. At the peak of the defensive enzyme activity of tobacco leaves treated with 10^8^ CFU/mL B4-7 fermentation broth, PAL activity was noted to be 106.56 U·g^-1^_·_min^-1^·FW, an increase of 33.42% and 81.42% compared to the disease control and healthy control respectively. CAT activity was 3.45×10^3^ U·g^-1^_·_min^-1^·FW, an increase of 121.15% and 69.19% relative to the disease control and healthy control. POD activity was 6.24×10^3^ U·g^-1^_·_min^-1^·FW, an increase of 106.22% and 36.75% compared to the disease and healthy controls. PPO activity was 135.56 U·g^-1^_·_min^-1^·FW, an increase of 162.66% and 40.84% compared to the disease and healthy controls. SOD activity reached 135.36 U·g^-1^_·_min^-1^·FW, an increase of 72.52% and 47.45% relative to the disease control and healthy control.

### 3.9 Effects of B4-7 on bacterial community structure in tobacco rhizosphere

**Figure 8:**
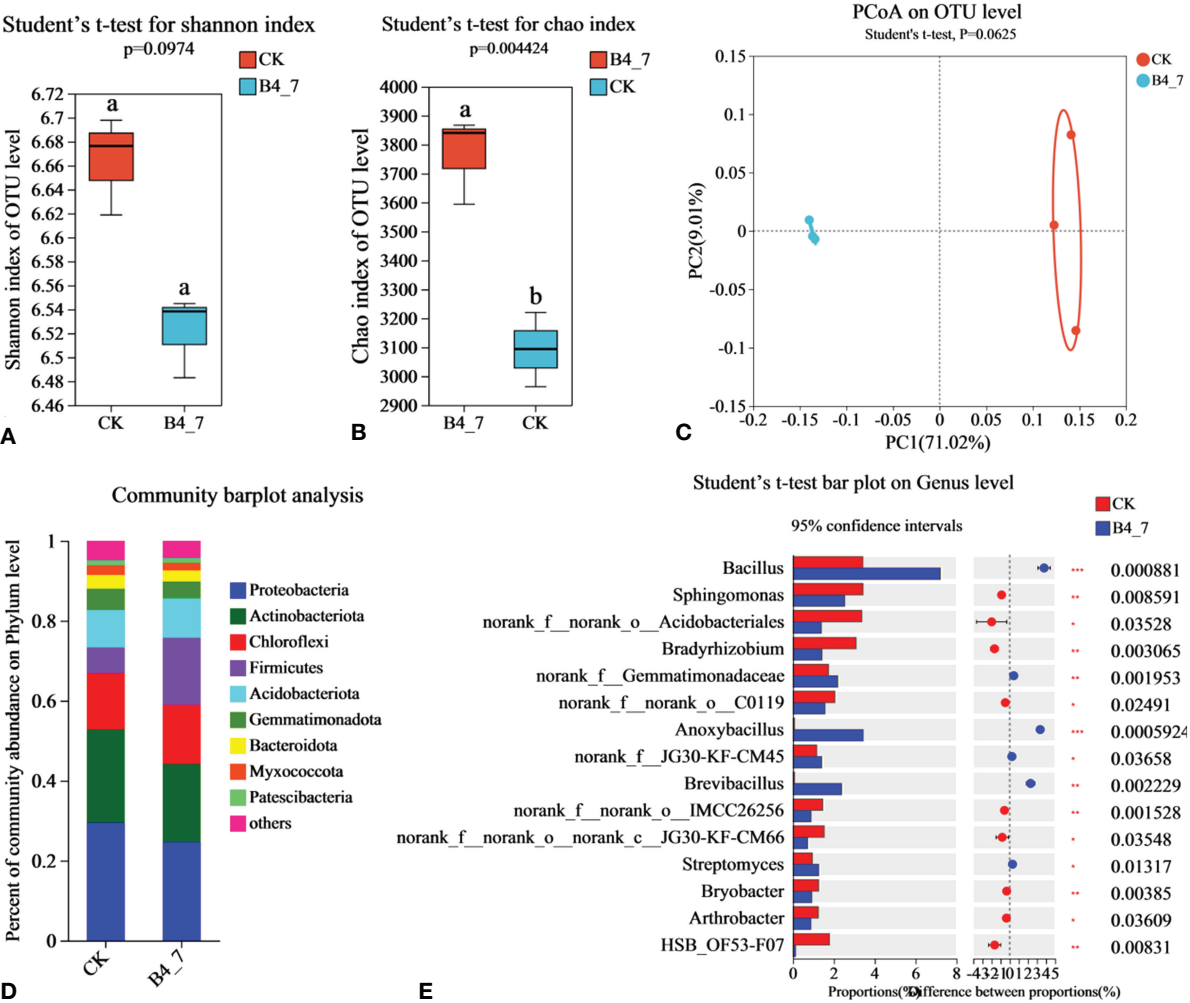
Effects of B4-7 on rhizosphere soil bacterial communities. Shannon diversity **(A)** and Chao richness **(B)** indices of bacteria community among the two soil samples. Different lowercase letters (Student’ s t-test, p < 0.05) indicate statistically significant differences between two groups. **(C)** The principal co-ordinates analysis (PCoA) and Beta diversity difference analysis. (Student’ s t-test, p < 0.05) indicates significant diversity between groups. **(D)** The relative abundance of bacterial phylum level among the two soil samples. **(E)** Hierarchical cluster analysis of 15 predominant bacterial communities among the two soil samples, asterisks (*) indicate significant differences determined by the Student’ s t test. ns, no significant; *p < 0.05; **p < 0.01; ***p <0.001.

To explore the influence of B4-7 on the tobacco field’s rhizosphere microbial community structure, total DNA was extracted from soil and analyzed through Illumina MiSeq. The effective sequences in the B4-7 treated soil and control soil (CK) bacteria sequencing results were 5,6157 and 3,7293, respectively. The sequencing depth was sufficient to represent the microbial community in these environments (**Supplementary Figure S1B**). According to Shannon and Chao indices, the abundance of bacteria in B4-7 increased significantly compared with CK, but there was no significant difference in diversity (**Figures 8A, B**). Based on the results of the PCoA with the Bray-Curtis measures, B4-7 and CK explained 80.03% of the total bacterial community variation in PC1 and PC2. Although there was significant separation on PC1 axis, combined with Beta diversity difference analysis, there was no significant (Student’ s t-test, p > 0.05) difference in species diversity between CK and B4-7 (**Figure 8C**). The composition of soil bacteria at the phylum level showed differences between the B4-7 treatment soil and the control soil. Abundance of phyla such as Proteobacteria, Actinobacteriota, Chloroflexi, Firmicutes, and Acidobacteriota exceeded 5% in both soils. Meanwhile, the abundance of Gemmatimonadota, Bacteroidota, Myxococcota, and Patescibacteria fell within the range of 1% to 5% in both soils. Comparatively, the relative abundances of Firmicutes and Myxococcota in the CK decreased by 67.73% and 11.13% respectively (**Figure 8D**). At the Genus level, the abundances of *Bacillus* (p < 0.001), norank_f:Gemmatimonadaceae (p < 0.01), *Anoxybacillus* (p < 0.001), *Brevibacillus* (p < 0.001), and *Streptomyces* (p < 0.05) significantly increased in the B4-7 compared to the CK (**Figure 8E**).

### 3.10 Effects of B4-7 on fungal community structure in tobacco rhizosphere

**Figure 9:**
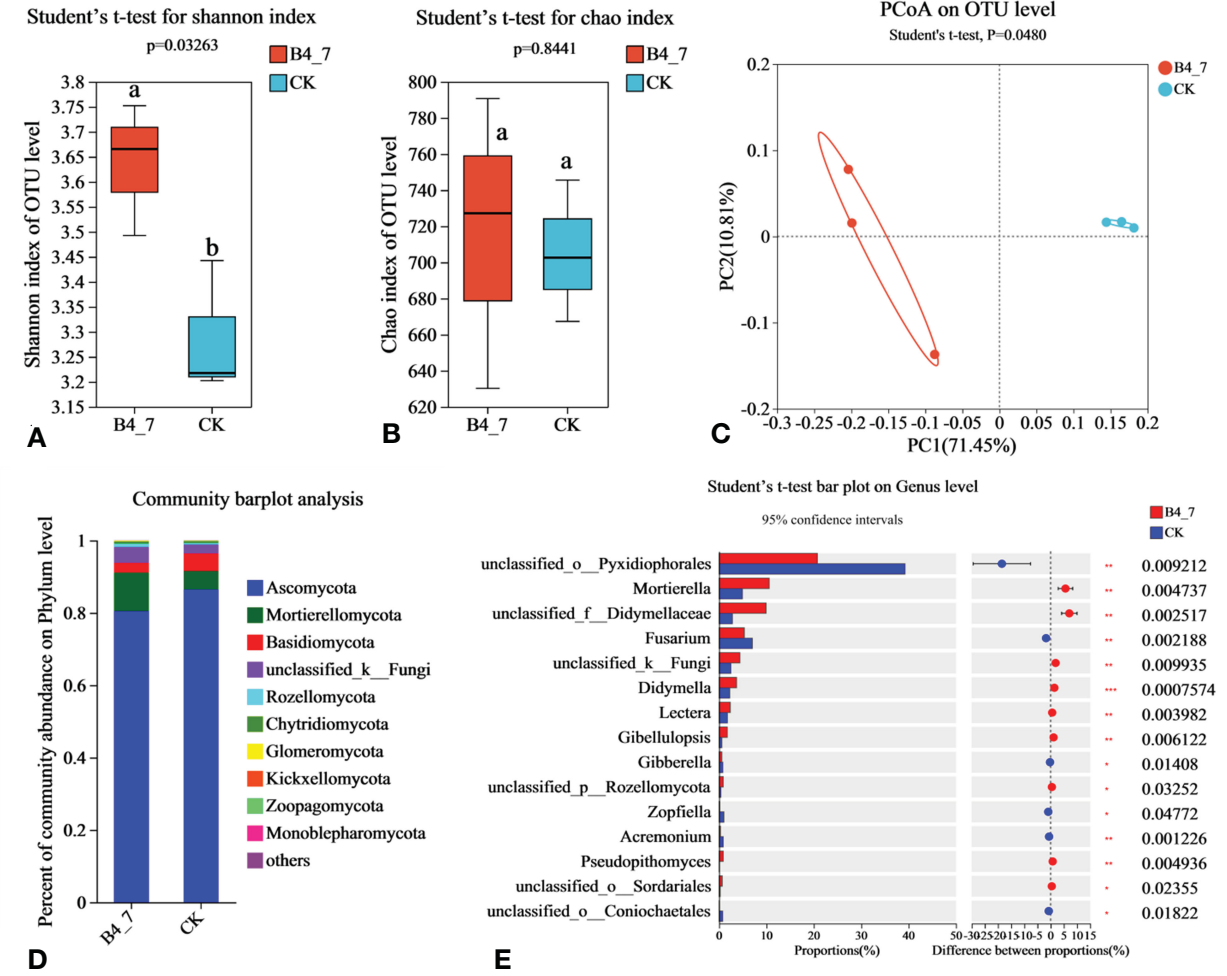
Effects of B4-7 on rhizosphere soil fungal community. Shannon diversity **(A)** and Chao richness **(B)** indices of fungal community among the two soil samples. Different lowercase letters (Student’ s t-test, p < 0.05) indicate statistically significant differences between groups. **(C)** The principal co-ordinates analysis (PCoA) and Beta diversity difference analysis. (Student’s t-test, p < 0.05) indicates significant diversity between groups. **(D)** The relative abundance of fungal phylum level among the two soil samples. **(E)** Hierarchical cluster analysis of 15 predominant fungal communities among the two soil samples, asterisks (*) indicate significant differences determined by the Student’ s t test. ns, no significant; *p < 0.05; **p < 0.01; ***p <0.001.

The effective sequences in the fungal sequencing results were 86939 and 86491 for the B4-7 and CK respectively. The sequencing depth was sufficient to represent the microbial community in these environments (**Supplementary Figure S1C**). According to Shannon and Chao indices, the diversity of fungal in B4-7 increased significantly compared with CK, but there was no significant difference in abundance (**Figures 9A, B**). Based on the results of the PCoA with the Bray-Curtis measures, B4-7 and CK explained 82.26% of the total bacterial community variation in PC1 and PC2. Combined with Beta diversity difference analysis, there was significant (Student′s t-test, p < 0.05) difference in species diversity between CK and B4-7 (**Figure 9C**). At the phylum, the abundance of Ascomycota, Mortierellomycota, Basidiomycota, and unclassified_k:Fungi exceeded 1%. The abundance of Rozellomycota, Chytridiomycota, Glomeromycota, Kickxellomycota, Zoopagomycota, Monoblepharomycota in both soils was less than 1% (**Figure 9D**). At the genus level, the abundances of *Mortierella* (p<0.01), unclassified_f:Didymellaceae (p<0.01), *Didymella* (p<0.001), *Lectera* (p<0.01), and *Gibellulopsis* (p<0.01) significantly increased in the B4-7 compared to CK. The abundance of unclassified_o:Pyxidiophorales (p<0.01) and *Fusarium* (p<0.01) were significantly reduced in B4-7 compared to CK (**Figure 9E**).

## 4 Discussion

The soil microbiome serves as a crucial bio-indicator of soil health (Schloter et al., 2018; Zhang et al., 2020b). Generally, a higher diversity and abundance index of a microbial community indicates a stronger capacity for recovery in response to various stressors. Studies have shown that the rhizosphere soil of tobacco plants in healthy tobacco fields exhibits higher microbial community diversity and abundance compared to fields with bacterial wilt disease (Wang et al., 2017). The findings of the present study align with these results, showing higher diversity and abundance of bacterial communities in suppressive soil (SS) compared to conducive soil (CS). At the phylum level, this study revealed that the abundance of Firmicutes in CS was significantly lower than in SS. A previous study by Lee et al., 2020, showed that the reduction of Firmicutes could be a key factor in the onset of bacterial wilt disease. Therefore, Firmicutes may be key species in maintaining SS against pathogen infection. At the phylum level, *Ralstonia* and *Pseudomonas* were concurrently enriched in the CS. Previous research has shown that *Pseudomonadaceae* forms part of the unique bacterial community present in diseased roots and exhibits antagonistic effects on pathogens (Schlatter et al., 2017). Therefore, it is possible that CS are recruiting *Pseudomonas* from the soil to combat pathogens. *Streptomyces* and *Bacillus* were found to be enriched in SS. *Streptomyces*, a class of gram-positive bacteria, are known for their production of abundant secondary metabolites (El-Shanshoury et al., 1996; Zhang et al., 2019). *Bacillus* spp., the most widely used biocontrol agents, are known for their effectiveness in controlling various plant diseases. *Bacillus* has an array of biocontrol mechanisms including secretion of hydrolytic enzymes and siderophores, production of secondary metabolites, and induction of systemic disease resistance in plants (Zheng et al., 2021; Chu et al., 2022; Qiu et al., 2022; Wang et al., 2022; Zhang et al., 2022; Liu et al., 2023). *Bacillus* can also improve plant growth by producing IAA, solubilizing phosphorus, resisting abiotic stress, and fixing nitrogen, making it a common plant growth-promoting bacteria (PGPB) (Sun et al., 2017; Wang et al., 2017; Jiao et al., 2020; Liu et al., 2023). Biocontrols based on *Bacillus* are typically more active than those based on other PGPB as *Bacillus* is more efficient at producing metabolites and forming spores, which improves its viability in commercially formulated products (Etesami et al., 2023). Thus, *Bacillus* in SS could serve as a critical microorganism in inhibiting the invasion of pathogens.

In this study, SS drawn from the tobacco rhizosphere was chosen as the source of bacterial samples. Forty distinct bacterial strains were isolated from the SS, six of which displayed inhibitory activity against *R. solanacearum*. Out of these, strain B4-7 candidly distinguished itself by exhibiting the highest degree of inhibitory effects on *R. solanacearum*, even under acidic conditions of pH 4.0. Given that the optimal pH range for bacterial growth is rather narrow and acidic environments are not conducive to their stable growth (Rousk et al., 2010; Tripathi et al., 2018; Schlatter et al., 2020), the acidophilic properties of B4-7 are pivotal in ensuring its effectiveness in acidic field soil. In greenhouse and field experiments, *B. velezensis* B4-7 demonstrated a significant control effect on TBW, and showed its potential to promote tobacco growth and boost yield. Similar results were found with *Bacillus*-fortified organic fertilizer BOF7, made from *B. amylolyticus* SQR-7, which recorded a control effect of 56.2% against TBW in the field and increased tobacco yields by 37.7% compared to the control (Yuan et al., 2016). *Bacillus* could suppressed the *R. solanacearum* populations in the rhizosphere soil (Li et al., 2017c). Results indicated that the concentration of *R. solanacearum* in the tobacco rhizosphere treated with B4-7 had significantly diminished compared with the controls.

The production of lipopeptide compounds as a mechanism of disease resistance in *Bacillus* is crucial for inhibiting or killing pathogenic bacteria (Chu et al., 2022). These lipopeptide compounds are known to possess tolerance to high temperatures, extreme acidity, extreme alkalinity, and ultraviolet radiation (Zhu et al., 2023). In this study, the lipopeptide crude extract of strain B4-7 exhibited resistance to high temperatures and demonstrated significant inhibitory effects on *R. solanacearum*. Genome analysis identified six gene clusters with 100% similarity that encode for the synthesis of secondary metabolites including surfactin, fengycin, bacillibactin, bacillaene, macrolactin E, and bacilysin. Further analyses using LC-MS confirmed the production of surfactin, bacillaene, macrolactin, bacilysin and fengycin derivatives by *B. velezensis* strain B4-7. MLNT has broad-spectrum antibacterial activity against both gram-positive and gram-negative bacteria (Li et al., 2017b). *Bacillus methylotrophicus* DR-08 has a significant control effect on tomato bacterial wilt through its antibacterial metabolites, such as difficidin, which inhibits the growth of most plant pathogens (Im et al., 2020). Fengycin inhibits mycelial growth by inducing deformation, oxidative damage, and mitochondrial dysfunction, while surfactin is known to increase the activity of biocontrol genes and enzymes in plants (Wang et al., 2020c). Nannan et al. (2021) found that *B. amyloliquefaciens*, *B. velezensis*, *B. pumilus*, and *B. subtilis* contained synthetic bacilysin gene clusters, and UHPLC-MS/MS analysis confirmed that bacilysin exhibited significant antagonistic activity against gram-negative pathogens. Wu et al. (2015) demonstrated that difficidin and bacilysin from *B. amyloliquefaciens* FZB42 have antibacterial activity against *Xanthomonas oryzae* of rice pathogen. The antibacterial compound C_15_-bacillomycin D isolated from soil bacterium *B. velezensis* NST6 has shown notable efficacy against *Staphylococcus epidermidis*, *S. aureus*, and methicillin-resistant *S. aureus* (Nam et al., 2021).

The plant’s protective response against pathogens is induced and coordinated by the salicylic acid (SA), jasmonic acid (JA), ethylene (ET) pathways, and defense-related enzymes (Wang et al., 2015; Peng et al., 2016; Qiu et al., 2022). When plants are exposed to various pathogenic organisms, defense enzymes such as POD, PPO, PAL, CAT, and SOD are induced. These enzymes play a role in the metabolism of disease-resistant secondary compounds (such as lignin, phenolic compounds, and plant defense hormones) (Richter et al., 2012; Shine et al., 2016), the metabolism of active oxygen species (Nakata et al., 2002; Singh et al., 2016), and directly inhibiting and killing pathogenic bacteria, thus enhancing the plants’ resistance to pathogens (Wei et al., 1992). Several studies have demonstrated a positive correlation between the activity of POD, PPO, PAL, CAT, SOD, and other enzymes and the disease resistance index, which represents a specific protective reaction against pathogens (Kashyap et al., 2021; Qiu et al., 2022). In this study, the application of *B. velezensis* B4-7 significantly enhanced the activity of various defense-related enzymes in tobacco plants, and this effect became more pronounced with increasing concentration. Combined with the results of Qiu et al. (2022) research, B4-7 can improve the disease resistance of plants by increasing a variety of defense enzymes.

The analysis of how biocontrol bacteria affect the soil microbial community is of utmost importance in understanding the mechanism behind the prevention and control of bacterial wilt by these biocontrol agents. Previous studies have demonstrated the efficacy of *B. amylolytica* ZM9, *B. amylolytica* Y4, and *Pseudomonas* sp. strain Y8 as biocontrol agents in inhibiting *R. solanacearum* and modulating tobacco rhizosphere microbial communities in field conditions (Wu et al., 2016; Li et al., 2021). B4-7 increased the abundance of bacteria and the diversity of fungi in rhizosphere soil. Furthermore, when compared to the control, it was observed that B4-7 significantly increased the abundance of Firmicutes and Mortierellomycota, while significantly reducing the abundance of Ascomycota and Basidiomycota. Additionally, studies have indicated that Ascomycota and Basidiomycota are known to include plant and insect pathogenic fungi (Muggia et al., 2020). Mortierellomycota contributes to the decomposition of organic matter and cellulose in soil, providing nutrients for plant growth (Purahong et al., 2016). At the genus level, the application of B4-7 significantly increased the abundance of *Bacillus* and *Streptomyces*. Furthermore, Wu et al. (2014) employed a bio-organic fertilizer plus soil amendment approach to increase the abundance of *Bacillus* and *Streptomyces* in the tobacco rhizosphere. It is possible that the significant difference observed in *Bacillus* abundance can be attributed to the steady growth of *B. velezensis* B4-7 in the tobacco rhizosphere soil, potentially attracting other *Bacillus* species. In bacterial wilt occurrences, there is often a high abundance of *Fusarium*, and the abundance of *Fusarium* and *R. solanacearum* are positively correlated (Su et al., 2020). Therefore, the significant reduction in *Fusarium* abundance due to B4-7 treatment can indirectly suggest a decrease in *R. solanacearum* population. By evaluating the microbial composition, it has been observed that B4-7 treatment can bring significant changes to the soil microbial community. This treatment increases the abundance and diversity of soil microorganisms, which may contribute to the improved resistance to tobacco bacterial wilt.

## 5 Conclusion

The study found significant differences in bacterial abundance and community composition between suppressive and conducive soils, indicating the importance of microbial communities in disease suppression. Specifically, the suppressive soil had a higher abundance of *Bacillus*, suggesting that *Bacillus* might be a key microorganism in suppressive soils. We successfully isolated a strain of *B. velezensis* B4-7 from the suppressive soil. Even under acidic conditions, this strain exhibited significant inhibitory activity against pathogenic bacteria. Greenhouse and field experiments were conducted to evaluate the effectiveness of B4-7 in controlling tobacco bacterial wilt (TBW). The results showed that B4-7 treatment led to a decrease in the disease index of TBW and reduced the number of rhizosphere pathogens. Additionally, it promoted tobacco growth and increased tobacco yield. B4-7 also enhanced the expression of various defense enzymes, further indicating its ability to increase tobacco’s disease resistance. Furthermore, genomic annotation and LC-MS analysis revealed that strain B4-7 had promising potential for the production of bioactive metabolites. In addition, B4-7 can increase the abundance of beneficial microorganisms, thereby improving the soil microecological environment. This suggests that B4-7 may be an important biocontrol agent (BCA) for the control of TBW.

## Data availability statement

The datasets presented in this study can be found in online repositories. The names of the repository/repositories and accession number(s) can be found below: NCBI, OM370806, OK330458 and CP080760.

## Author contributions

X-JM: Data curation, Investigation, Methodology, Writing – original draft. L-QW: Investigation, Methodology, Writing – original draft. B-GM: Investigation, Methodology, Writing – original draft. X-HW: Investigation, Methodology, Writing – original draft. YZ: Writing – review & editing. Z-XS: Funding acquisition, Project administration, Supervision, Writing – review & editing. Y-YL: Funding acquisition, Project administration, Writing – review & editing.
