# Supplementary material for: Screening, identification and evaluation of an acidophilic strain of Bacillus velezensis B4-7 for the biocontrol of tobacco bacterial wilt
Source: Front Plant Sci. 2024 May 1;15:1360173. doi: 10.3389/fpls.2024.1360173 (PMC11094357; doi:10.3389/fpls.2024.1360173)
Supplement: Supplementary file 1 [file DataSheet_1.docx]

**Table S1**. Prediction information of BCGs in strain B4-7 genome

| Type | Start | End | Similar Cluster | Similarity |
| --- | --- | --- | --- | --- |
| NRPS | 320450 | 384427 | surfactin | 82% |
| PKS-like | 921097 | 962.341 | butirosin A/B | 7% |
| transAT-PKS | 1381126 | 1468961 | macrolactin H | 100% |
| transAT-PKS, T3PKS | 1688490 | 1798064 | bacillaene | 100% |
| NRPS, transAT-PKS | 1862797 | 1997107 | fengycin | 100% |
| transAT-PKS-like | 2267032 | 2373214 | difficidin | 100% |
| NRPS, RiPP-like | 2997918 | 3049709 | bacillibactin | 100% |
| other | 3586019 | 3627437 | bacilysin | 100% |
| terpene | 1047220 | 1064628 | — | — |
| lanthipeptide-class-ii | 1185618 | 1214506 | — | — |
| terpene | 2025745 | 2047628 | — | — |
| T3PKS | 2110946 | 2152046 | — | — |


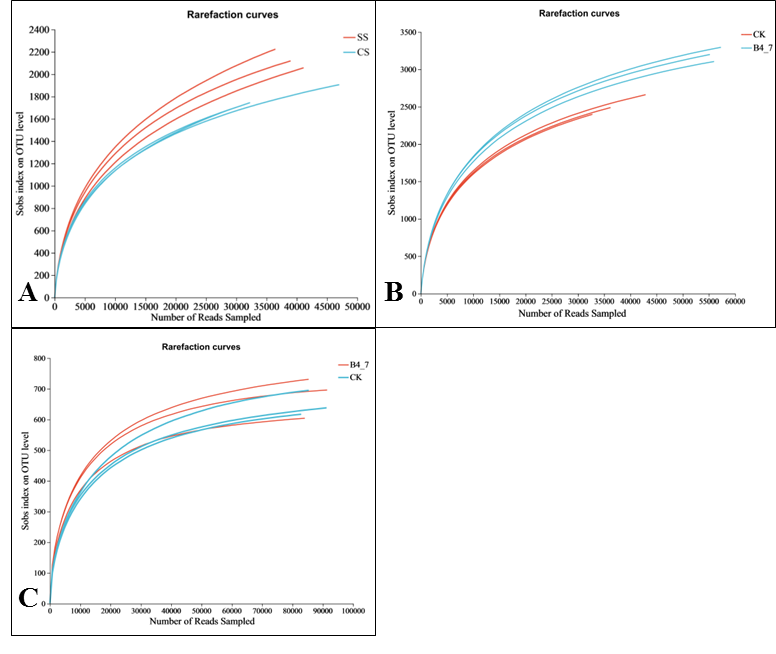


**Figure S1.** Rarefaction curve analysis of bacterial/ fungal species richness in rhizosphere soil samples. (A) Dilution curves of bacterial species richness in rhizosphere soil samples of suppressive and conducive soil. (B) Dilution curves of bacterial species richness in rhizosphere soil samples of B4-7 treatment soil and Control (CK) soil. C. Dilution curves of fungal species richness in rhizosphere soil samples of B4-7 treatment soil and Control (CK) soil


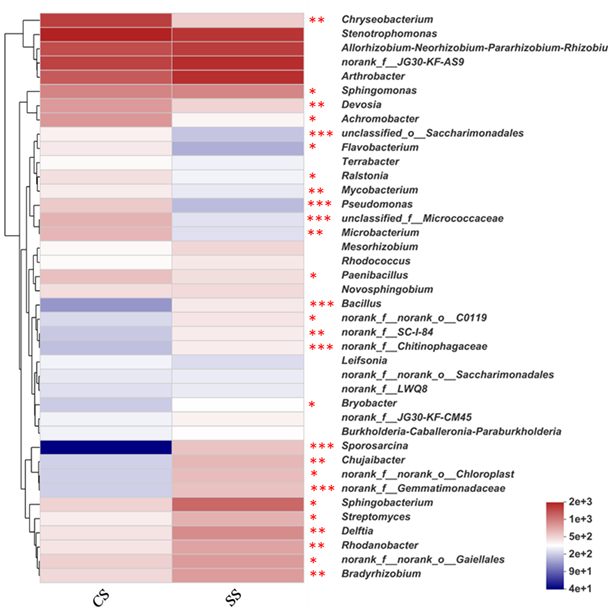


**Figure S2.** Heatmap of 40 predominant bacterial genus communities among the SS and CS. Asterisks (*) indicate significant differences determined by the Student’ s t-test. ns, no significant; *p < 0.05; **p < 0.01; ***p < 0.001


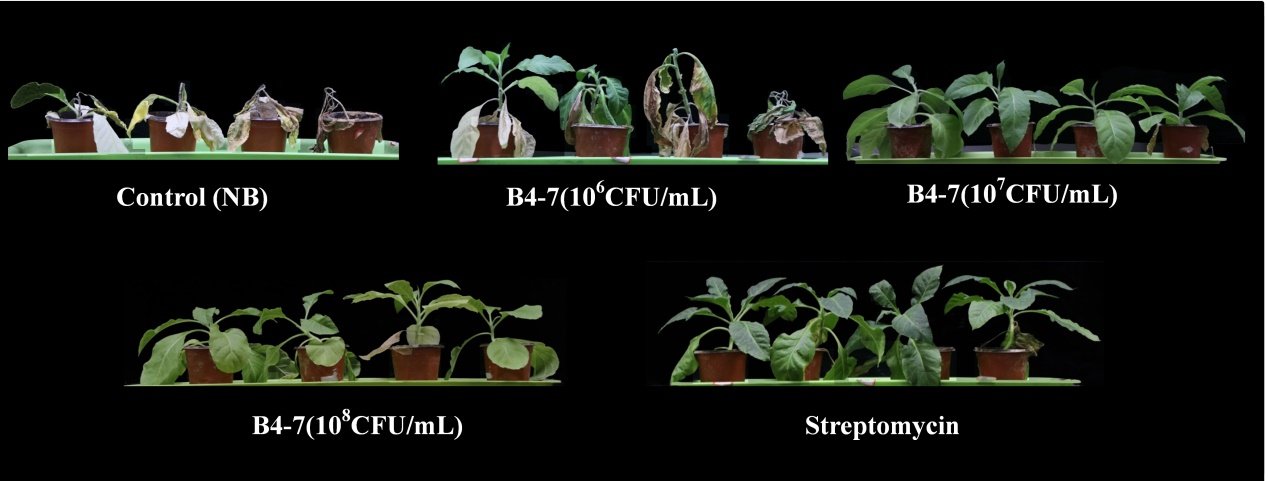


**Figure S3.** Symptoms of tobacco bacterial wilt (TBW) on seedlings 21d after inoculation. 10^6^CFU/mL: 10^6^CFU/mL B4-7 fermentation broth treatment. 10^7^CFU/mL: 10^7^CFU/mL B4-7 fermentation broth treatment. 10^8^CFU/mL: 10^8^CFU/mL B4-7 fermentation broth treatment. NB: NB treatment. Streptomycin: Streptomycin (100 mg/L) treatment.


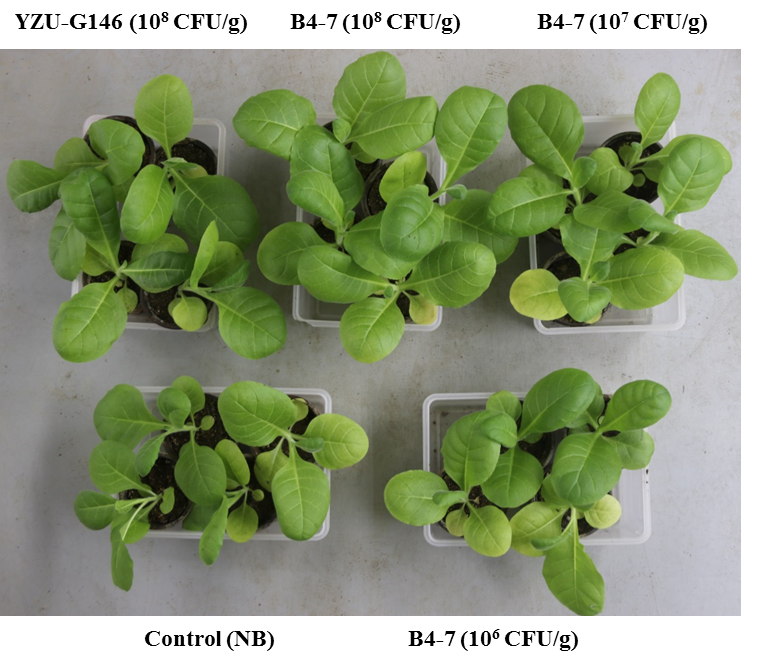


**Figure S4.** Views of tobacco seedlings grown with different treatments in 30d. The bacterial fermentation broth with different concentrations was mixed with soil. B4-7(10^8^CFU/g) : Concentration of B4-7 in soil is 10^8^CFU/g. B4-7(10^7^CFU/g): Concentration of B4-7 in soil is 10^7^CFU/g. B4-7(10^6^CFU/g): Concentration of B4-7 in soil is 10^6^CFU/g. YZU-G146(10^8^CFU/g): Concentration of G146 in soil is 10^8^CFU/g. Control (NB): NB was mixed with soil.

**Figure S5.** Sensitivity detection of primers of qPCR. A. qPCR amplification curves of 10-fold serial dilutions; B. qPCR melting curves of 10-fold serial dilutions; C. Electrophoretic analysis of conventional PCR products by 10-fold serial dilutions, a~h: The initial concentration of plasmids was diluted by 10^0^~10^7^; D. qPCR standard curves of plasmid of 10-fold serial dilutions.


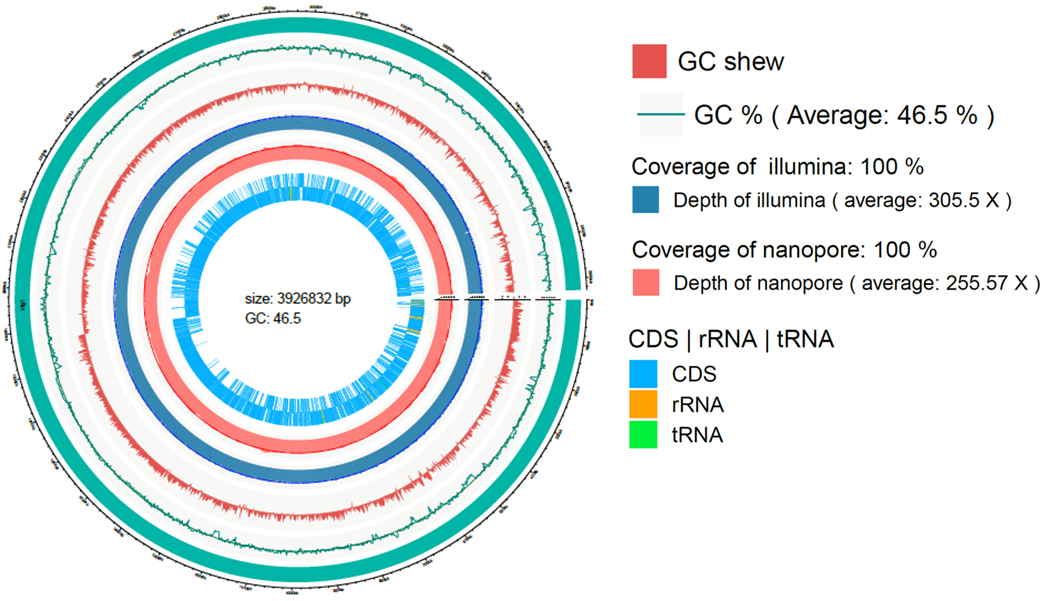


**Figure S6.** Circular map of strain B4-7 genome


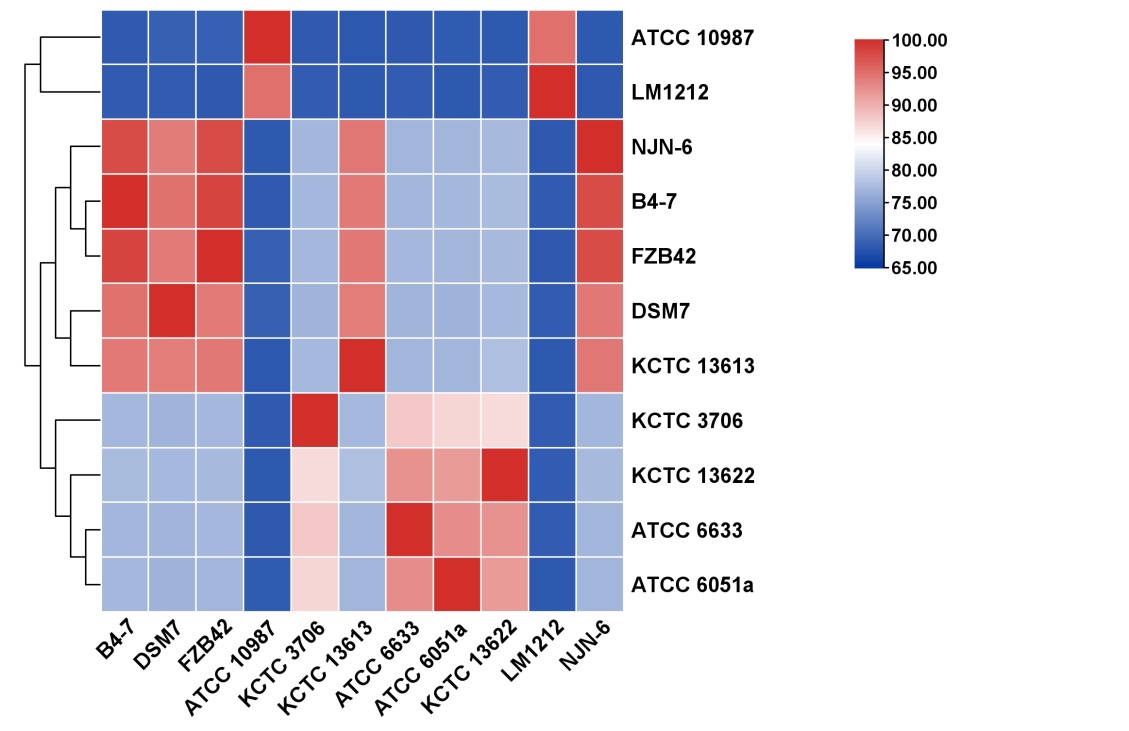


**Figure S7.** Average Nucleotide Identity (ANI, %) of B4-7 and other type strains. Genome comparison between B4-7 and 10 homologous bacterial strains. DSW7: *Bacillus amyloliquefaciens* DSM7; FZB42: *B.velezensis* FZB42; ATCC 10987: *B.cereus* ATCC 10987; KCTC 3706: *B.mojavensis* KCTC 3706; KCTC 13613: *B.siamensis* KCTC 13613; ATCC 6633: *B.spizizenii* ATCC 6633; ATCC 6051a: *B.subtilis* ATCC 6051a; KCTC 13622: *B.tequilensis* KCTC 13622; LM1212: *B.thuringiensis* LM1212; *B.velezensis* NJN-6. Draw heatmap by Tbtools.


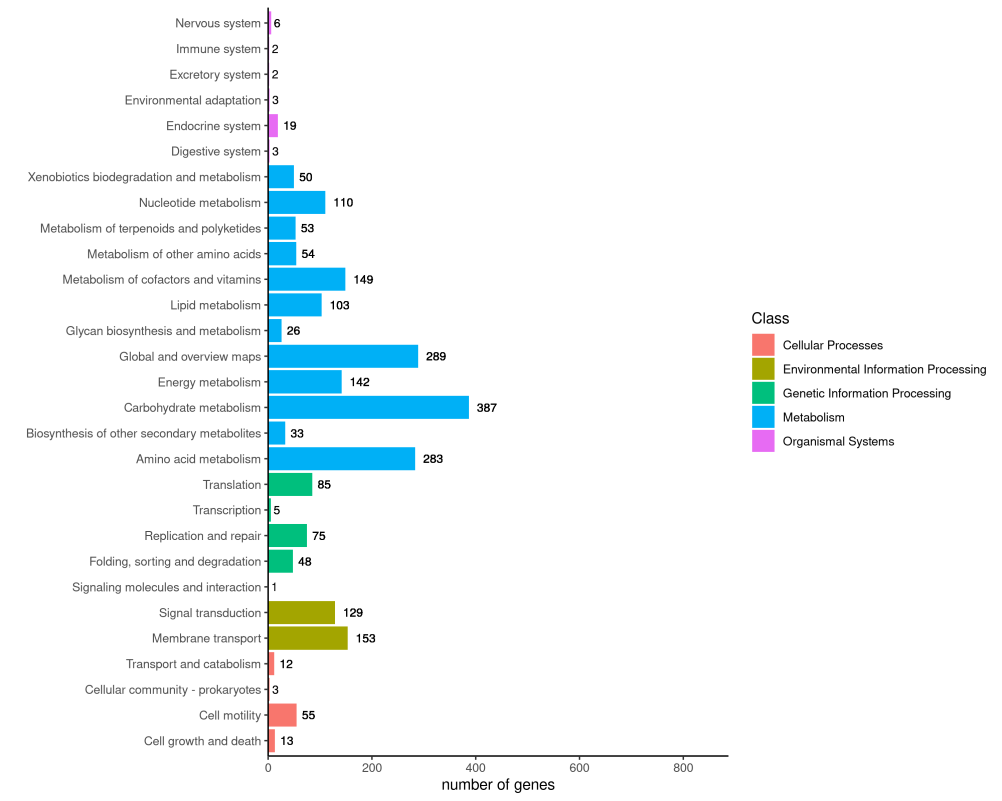


**Figure S8.** KEGG passageway annotation of the strain B4-7 genome


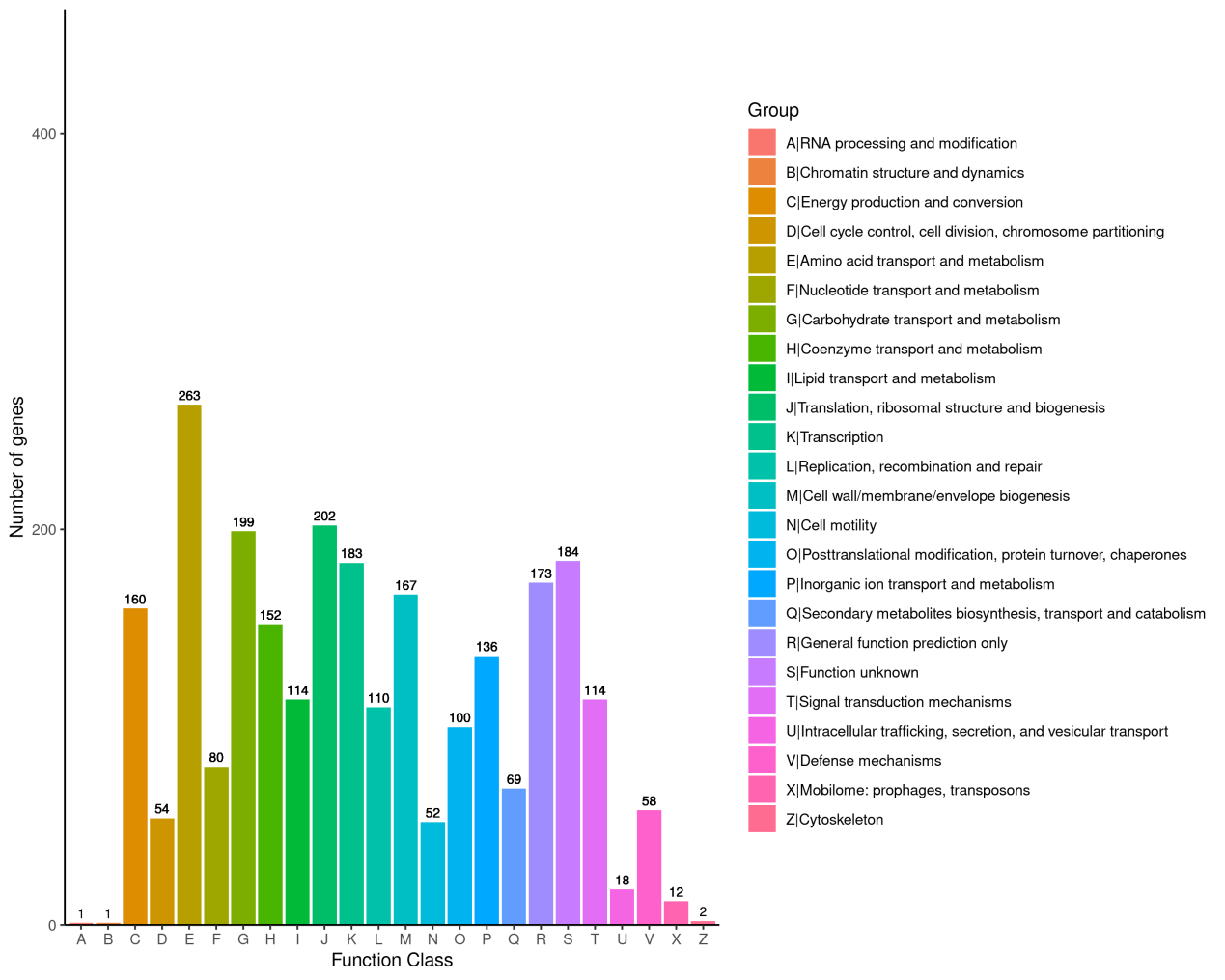


**Figure S9.** COG annotation of the strain B4-7 genome


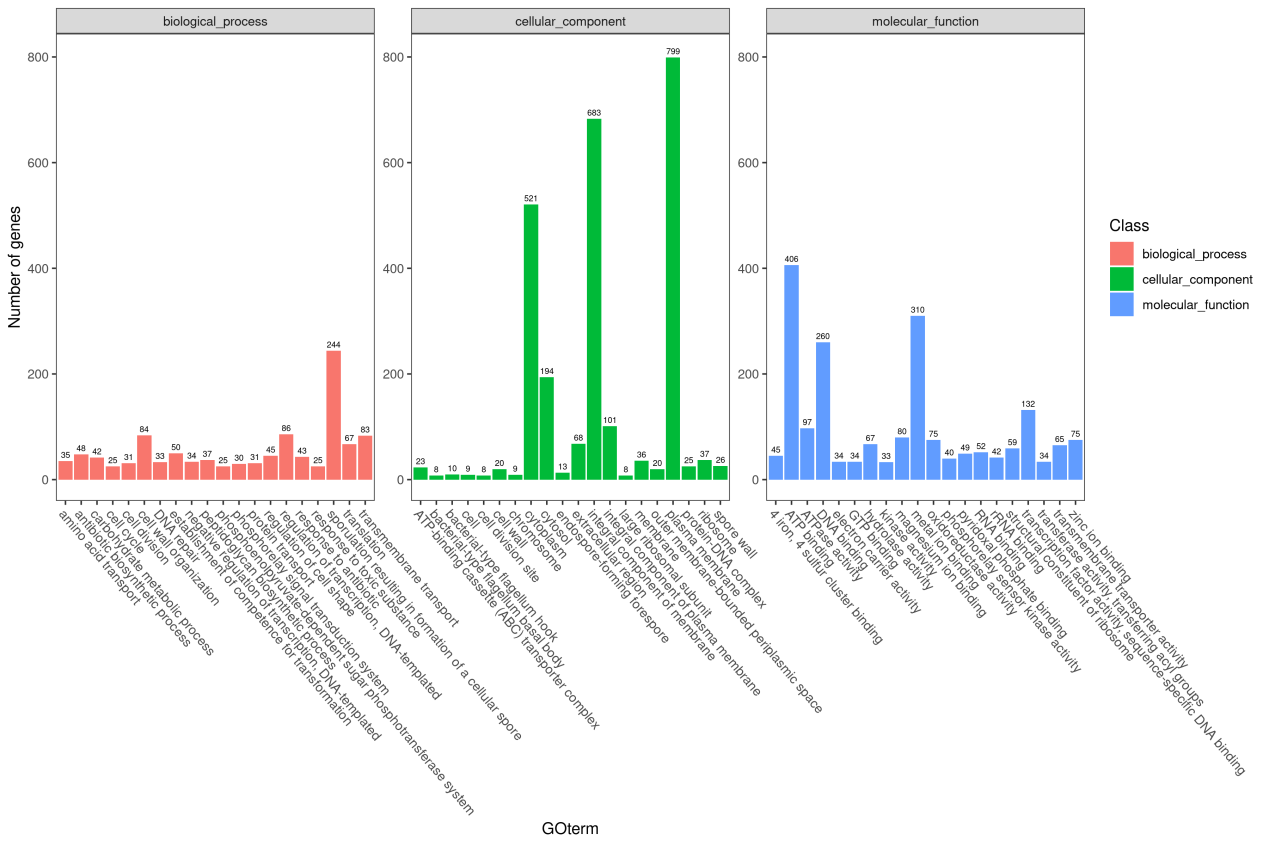


**Figure S10.** GO annotation of the strain B4-7 genome

**Figure S11.** Mass spectrogram of components in antimicrobial active substances of strain B4-7. (a) Macrolactin A; (b) Microlactin E; (c) Bacillaene; (d) Surfactin A; (e) Surfactin B; (f) Surfactin C; (g) Difficidin; (h) Bacilysin; (i) Pachymic acid; (j) Benzoylmesaconine; (k) Troxerutin; (l) 3,4',5-Trimethoxy-trans-stilbene.
