# Supplementary material for: Screening, identification and evaluation of an acidophilic strain of Bacillus velezensis B4-7 for the biocontrol of tobacco bacterial wilt
Source: Front Plant Sci. 2024 May 1;15:1360173. doi: 10.3389/fpls.2024.1360173 (PMC11094357; doi:10.3389/fpls.2024.1360173)
Supplement: Supplementary file 2 [file Table_1.docx]

**Supplementary Materials and methods**

# S1. PCR amplification and high-throughput sequencing of soil microorganism

1.1 DNA extraction and PCR amplification

Total microbial genomic DNA was extracted from soil samples using the E.Z.N.A.® soil DNA Kit (Omega Bio-tek, Norcross, GA, U.S.) according to manufacturer’s instructions. The quality and concentration of DNA were determined by 1.0% agarose gel electrophoresis and a NanoDrop2000 spectrophotometer (Thermo Scientific, United States) and kept at -80 °C prior to further use. The bacterial 16S rRNA/ fungal ITS gene were amplified with primer pairs 338F (5'-ACTCCTACGGGAGGCAGCAG-3') and 806R(5'-GGACTACHVGGGTWTCTAAT-3')/ ITS1F (5'-CTTGGTCATTTAGAGGAAGTAA-3') and ITS2R (5'-GCTGCGTTCTTCATCGATGC-3') by T100 Thermal Cycler PCR thermocycler (BIO-RAD, USA). The PCR reaction mixture including 4 μL 5 × Fast Pfu buffer, 2 μL 2.5 mM dNTPs, 0.8 μL each primer (5 μM), 0.4 μL Fast Pfu polymerase, 10 ng of template DNA, and ddH_2_O to a final volume of 20 µL. PCR amplification cycling conditions were as follows: initial denaturation at 95 °C for 3 min, followed by 27 cycles of denaturing at 95 °C for 30 s, annealing at 55 °C for 30 s and extension at 72 °C for 45 s, and single extension at 72 °C for 10 min, and end at 4 °C. The PCR product was extracted from 2% agarose gel and purified using the PCR Clean-Up Kit (YuHua, Shanghai, China) according to manufacturer’s instructions and quantified using Qubit 4.0 (Thermo Fisher Scientific, USA) .

1.2 Data processing

Raw FASTQ files were de-multiplexed using an in-house perl script, and then quality-filtered by fastp version 0.19.6 (Chen et al., 2018) and merged by FLASH version 1.2.7 (Magoč et al., 2011) with the following criteria: (i) the reads were truncated at any site receiving an average quality score of <20 over a 50 bp sliding window, and the truncated reads shorter than 50 bp were discarded, reads containing ambiguous characters were also discarded; (ii) only overlapping sequences longer than 10 bp were assembled according to their overlapped sequence. The maximum mismatch ratio of overlap region is 0.2. Reads that could not be assembled were discarded; (iii) Samples were distinguished according to the barcode and primers, and the sequence direction was adjusted, exact barcode matching, 2 nucleotide mismatch in primer matching. Then the optimized sequences were clustered into operational taxonomic units (OTUs) using UPARSE 7.1 (Edgar et al., 2013; Stackebrandt et al., 1994) with 97% sequence similarity level. The most abundant sequence for each OTU was selected as a representative sequence. To minimize the effects of sequencing depth on alpha and beta diversity measure, the number of 16S rRNA gene sequences from each sample were rarefied to 20,000, which still yielded an average Good’s coverage of 99.09%.

The taxonomy of each OTU representative sequence was analyzed by RDP Classifier version 2.2 (Wang et al., 2007) against the 16S rRNA/ITS gene databases using confidence threshold of 0.7. The metagenomic function was predicted by Phylogenetic Investigation of Communities by Reconstruction of Unobserved States (PICRUSt2) (Douglas et al., 2020) based on OTU representative sequences. Difference expression analysis are carried out by the edgeR package.

16S rRNA gene databases: Silva（Release138 http://www.arb-silva.de）and RDP (https://rdp.cme.msu.edu/). ITS gene databases: Unite (Release 8.0 http://unite.ut.ee/index.php）

1.3 Statistical Analysis

Bioinformatic analysis of the soil microbiota was carried out using the Majorbio Cloud platform (https://cloud.majorbio.com). Based on the OTUs information, rarefaction curves and alpha diversity indices including, Chao richness and Shannon index were calculated with Mothur v1.30.1 (Schloss., et al., 2009). The similarity among the microbial communities in different samples was determined by principal coordinate analysis (PCoA) based on Bray-curtis dissimilarity using Vegan v2.5-3 package. The Student′s t - test of Beta diversity difference analysis was used to assess the percentage of variation explained by the treatment along with its statistical significance using R software (version 3.3.1) and distance matrix Qiime (V1.9.1). STAMP software was used to analyze the species abundance difference between groups, Student’ s t - test was used to analyze the species abundance difference between different soil types at phylum and genus level, and FDR was used for multiple test correction.

# S2. Quantitative real-time (qPCR) detection method

Total DNA was extracted from the tobacco rhizosphere soil using a soil microbial DNA kit (Solarbio), and the concentration and purity of the DNA were analyzed using an ultramicro spectrophotometer. The DNA was then stored at -20°C for future use. For the detection of *R. solanacearum*, a specific primer combination of 199F (5'-AGTAACTCGGCTGTTTTTTT-3') and 199R (5'-TATTGCTTGACCTATAA-3') (Freitas et al., 2021) was selected. The PCR amplification reaction system included 2× Taq Master Mix (12.5 μL), DNA template (1.0 μL), forward and reverse primers (1.0 μL each), and ddH_2_O to make a final volume of 25.0 μL. The amplification procedure consisted of pre-denaturation at 95°C for 2 min, followed by 35 cycles at 95°C for 30 s, 55°C for 30 s, and 72°C for 60 s. A final extension step was performed at 70°C for 8 min, followed by storage at 16°C. The PCR products were then detected and recovered using 1% agarose gel electrophoresis. The recovered products were cloned into the pMD18-T vector (TaKaRa) and transformed into *Escherichia coli* DH5α (WEIDI Co., Ltd; Shanghai municipality, China) competent cells. Plasmid DNA was extracted after blue and white spot screening, and nucleic acid sequencing was performed by Wuhan Huada Gene Co., Ltd. For quantitative analysis of *R. solanacearum*, plasmid standards with eight concentration gradients (10^1^~10^8^ copies/μL) were prepared by 10-fold dilution. For the real-time fluorescence quantitative PCR reaction, the system included 1.0 μL of upstream primer, 1.0 μL of downstream primer, 10.0 μL of qPCR SYBR Green Master Mix, 1.0 μL of DNA template, and 20.0 μL of ddH_2_O. The amplification procedure consisted of pre-denaturation at 96°C for 1 min, followed by 35 cycles at 95°C for 15 s, 55°C for 35 s, 72°C for 30 s, and a final extension step at 70°C for 10 min. The reaction was then stored at 4°C. The melting curve steps included heating to 95°C for 15 s, cooling to 60°C for 1 min, then re-heating to 95°C for 15 s. After the reaction, the amplification curve and melting curve were confirmed. The Ct values corresponding to the amplification curve were used to draw a standard curve. The soil DNA samples were used as templates for qPCR amplification using the specific primer combination 199F/199R. Each sample was repeated five times, and the content of *R. solanacearum* in the soil was calculated based on the Ct values obtained.

# S3. Liquid Chromatography-Mass Spectrometry (LC-MS) detection method

The lipopeptide secondary metabolites of strain B4-7 were dissolved in a small amount of methanol and then diluted 10 times and 100 times, respectively. These diluted solutions were filtered through a microporous filter to remove any particulate matter and ensure sample purity. The filtered solutions were then subpacked into special sample bottles that are suitable for LC-MS analysis. The LC-MS analysis was conducted using a Kinetex F5 column with dimensions of 100 mm × 2.1 mm and a particle size of 2.6 μm. The mobile phase consisted of ultra-pure water (mobile phase A) and LC-MS grade acetonitrile (mobile phase B). Gradient elution was performed according to the following conditions: 0~2 min: 5% mobile phase B; 2-8 min: 5~60% mobile phase B; 8-20 min: 60-95% mobile phase B; 20~25 min: 95% mobile phase B; 25~25.01 min: 95~5% mobile phase B; 25.01~27 min: 5% mobile phase B. The sample size injected into the LC-MS system was 10 μL, and the flow rate was set at 300 μL/min. The detection wavelength for analysis was 254 nm. For mass spectrometry analysis, an ESI (electrospray ionization) ion source was utilized in both positive and negative ion modes. The ion spray voltage was set at 5000 V, and the ion source temperature was maintained at 500°C. The column temperature was kept constant at 40°C during the analysis. The mass scanning range (m/z) was set from 100 to 1250 to cover the relevant mass range for lipopeptide identification. To interpret and analyze the initial LC-MS data, SCIEX OS 1.7.0 software was used. By comparing the obtained data with existing information in the database, the information regarding the main components of the lipopeptide active substances produced by strain B4-7 was obtained (Meng et al., 2023; Jin et al., 2020).

**References**

Chen, S. F., Zhou, Y. Q., Chen, Y. R. & Gu, J. (2018). fastp: an ultra-fast all-in-one FASTQ preprocessor. *Bioinformatics*, *34* (17), pp.i884-i890. doi:10.1093/bioinformatics/bty560

Douglas G. M., Maffei V. J, Zaneveld J. R, Yurgel S. N, Brown J. R, Taylor C. M, et al. (2020). PICRUSt2 for prediction of metagenome functions[J]. *Nature Biotechnology*, *38*: 685–688. doi:10.1038/s41587-020-0548-6

Edgar, Robert. C. (2013). UPARSE: highly accurate OTU sequences from microbial amplicon reads.[J]. *Nature Methods*, *10* (10):996‐998. doi:10.1038/nmeth.2604

Freitas, R. G., Hermenegildo, P. S., Cascardo, R. S., Guimarães, L. M. S., Santos, S. A., Badel, J. L., et al. (2021). Validation and use of a qPCR protocol to quantify the spread of *Ralstonia solanacearum* in susceptible and resistant eucalypt plants. *Plant Pathology*, *70* (7), 1708-1718. doi:10.1111/ppa.13406

Jin, P. F., Wang, H. N., Tan, Z., Xuan, Z., Dahar, G. Y., Li, Q. X., et al. (2020). Antifungal mechanism of bacillomycin D from *Bacillus velezensis* HN-2 against Colletotrichum gloeosporioides Penz. *Pestic. Biochem. Physiol. 163*, 102–107. doi:10.1016/j.pestbp.2019.11.004

Meng, X-J., Medison, R. G., Cao, S., Wang, L-Q., Cheng, S., Tan, L-T., et al. (2023). Isolation, identification, and biocontrol mechanisms of endophytic *Burkholderia vietnamiensis* C12 from *Ficus tikoua* Bur against *Rhizoctonia solani*. *Biological Control, 178*, 105132. doi:10.1016/j.biocontrol.2022.105132

Magoč, T. and FLASH, S.S. (2011). Fast length adjustment of short reads to improve genome assemblies. *Bioinformatics*, *27* (21): 2957‐2963. doi:10.1093/bioinformatics/btr507

Stackebrandt E., Goebel B. M. (1994). Taxonomic Note: A Place for DNA-DNA Reassociation and 16S rRNA Sequence Analysis in the Present Species Definition in Bacteriology [J]. *Int.j.syst.bacteriol, 44* (4):846-849. doi:10.1099/00207713-44-4-846

Schloss, P. D., Westcott, S. L., Ryabin, T., Hall, J. R., Hartmann, M., Hollister, E. B., et al. (2009). Introducing mothur: open-source, platform-independent, community-supported software for describing and comparing microbial communities. *Applied and environmental microbiology*, *75* (23), pp.7537-7541. doi:10.1128/AEM.01541-09

Wang, Q., Garrity, G. M., Tiedje, J. M., & Cole, J. R. (2007). Naive Bayesian classifier for rapid assignment of rRNA sequences into the new bacterial taxonomy. Applied and environmental microbiology, 73(16), 5261-5267. doi: 10.1128/AEM.00062-07.
